# Psychotherapies for eating disorders: findings from a rapid review

**DOI:** 10.1186/s40337-023-00886-w

**Published:** 2023-10-04

**Authors:** Haley Russell, Phillip Aouad, Anvi Le, Peta Marks, Danielle Maloney, National Eating Disorder Research Consortium, Phillip Aouad, Sarah Barakat, Robert Boakes, Leah Brennan, Emma Bryant, Susan Byrne, Belinda Caldwell, Shannon Calvert, Bronny Carroll, David Castle, Ian Caterson, Belinda Chelius, Lyn Chiem, Simon Clarke, Janet Conti, Lexi Crouch, Genevieve Dammery, Natasha Dzajkovski, Jasmine Fardouly, John Feneley, Nasim Foroughi, Mathew Fuller-Tyszkiewicz, Anthea Fursland, Veronica Gonzalez-Arce, Bethanie Gouldthorp, Kelly Griffin, Scott Griffiths, Ashlea Hambleton, Amy Hannigan, Mel Hart, Susan Hart, Phillipa Hay, Ian Hickie, Francis Kay-Lambkin, Ross King, Michael Kohn, Eyza Koreshe, Isabel Krug, Jake Linardon, Randall Long, Amanda Long, Sloane Madden, Sarah Maguire, Danielle Maloney, Peta Marks, Siân McLean, Thy Meddick, Jane Miskovic-Wheatley, Deborah Mitchison, Richard O’Kearney, Shu Hwa Ong, Roger Paterson, Susan Paxton, Melissa Pehlivan, Genevieve Pepin, Andrea Phillipou, Judith Piccone, Rebecca Pinkus, Bronwyn Raykos, Paul Rhodes, Elizabeth Rieger, Karen Rockett, Sarah Rodan, Janice Russell, Haley Russell, Fiona Salter, Susan Sawyer, Beth Shelton, Urvashnee Singh, Sophie Smith, Evelyn Smith, Karen Spielman, Sarah Squire, Juliette Thomson, Marika Tiggemann, Stephen Touyz, Ranjani Utpala, Lenny Vartanian, Sabina Vatter, Andrew Wallis, Warren Ward, Sarah Wells, Eleanor Wertheim, Simon Wilksch, Michelle Williams, Stephen Touyz, Sarah Maguire

**Affiliations:** 1 InsideOut Institute, Central Clinical School, Faculty of Medicine and Health, Level 2, Charles Perkins Centre (D17), University of Sydney, Sydney, NSW 2006 Australia; 2 Sydney Local Health District, New South Wales Health, Sydney, Australia; 3 Healthcare Management Advisors, Melbourne, Australia; 4 InsideOut Institute, Central Clinical School, Faculty of Medicine and Health, University of Sydney, Sydney, NSW Australia; 5 School of Psychology, Faculty of Science, University of Sydney, Sydney, NSW Australia; 6 School of Psychology and Public Health, La Trobe University, Victoria, Australia; 7 School of Psychology, Western Australia, Perth, Australia; 8 Eating Disorders Victoria, Victoria, Australia; 9 Perth, WA Australia; 10 Medicine, Dentistry and Health Sciences, University of Melbourne, Victoria, Australia; 11 School of Life and Environmental Sciences, University of Sydney, Sydney, NSW Australia; 12 Eating Disorders Queensland, Brisbane, QLD Australia; 13 Westmead Hospital, Sydney, NSW Australia; 14 Translational Health Research Institute, Western Sydney University, Sydney, NSW Australia; 15 Brisbane, QLD Australia; 16 School of Psychology, University of New South Wales, Sydney, NSW Australia; 17 New South Wales Health, New South Wales, Australia; 18 School of Psychology, Faculty of Health, Deakin University, Victoria, Australia; 19 School of Population Health, Faculty of Health Sciences, Curtain University, Perth, Australia; 20 Hollywood Clinic, Ramsay Health Care, Perth, Australia; 21 Melbourne School of Psychological Sciences, University of Melbourne, Victoria, Australia; 22 Queensland Eating Disorder Service, Brisbane, QLD Australia; 23 Hunter New England Local Health District, New South Wales, Australia; 24 St Vincent’s Hospital Network Local Health District, Sydney, NSW Australia; 25 Brain and Mind Centre, University of Sydney, Sydney, Australia; 26 School of Medicine and Public Health, University of Newcastle, New South Wales, Australia; 27 Westmead Hospital, University of Sydney, Sydney, Australia; 28 College of Medicine and Public Health, Flinders University, Adelaide, SA Australia; 29 Exchange Consultancy, Redlynch, NSW Australia; 30 Eating Disorders Service, Children’s Hospital at Westmead, Sydney, NSW Australia; 31 Clinical Excellence Queensland, Mental Health Alcohol and Other Drugs Branch, Brisbane, QLD Australia; 32 College of Health and Medicine, Australian National University, Canberra , ACT Australia; 33 ADHD and BED Integrated Clinic, Melbourne, VIC Australia; 34 Department of Psychology and Counselling, La Trobe University, Victoria, Australia; 35 School of Health and Social Development, Faculty of Health, Deakin University, Geelong, VIC Australia; 36 Swinburne Anorexia Nervosa (SWAN) Research Group, Centre for Mental Health, School of Health Sciences, Swinburne University, Victoria, Australia; 37 Children’s Health Queensland Hospital and Health Service, Brisbane, QLD Australia; 38 Centre for Clinical Interventions, Western Australia Health, Perth, WA Australia; 39 Central Clinical School Brain and Mind Research Institute, University of Sydney, New South Wales, Sydney, Australia; 40 Ramsay Health Care, Perth, Australia; 41 Department of Paediatrics, The University of Melbourne, Melbourne, Australia; 42 National Eating Disorders Collaboration, Victoria, Australia; 43 The Hollywood Clinic Hollywood Private Hospital, Ramsey Health, Perth, Australia; 44 Sydney, NSW Australia; 45 The Butterfly Foundation, Sydney, Australia; 46 College of Education, Psychology and Social Work, Flinders University, Adelaide, SA Australia; 47 Eating Disorder Service, The Sydney Children’s Hospital Network, Westmead Campus, Sydney, Australia; 48 Department of Psychiatry, University of Queensland, Brisbane, Australia; 49 University of Tasmania, Tasmania, Australia; 50 Royal Hobart, Tasmanian Health Service, Hobart, TAS Australia

**Keywords:** Eating disorders, Psychotherapies, Therapies, Treatment, Intervention, Behavioural therapies cognitive behavioural therapy, Dialectical behavioural therapy, Family-based therapy

## Abstract

**Background:**

Psychotherapy is considered central to the effective treatment of eating disorders—focusing on behavioural, psychological, and social factors that contribute to the illness. Research indicates psychotherapeutic interventions out-perform placebo, waitlist, and/or other treatments; but, outcomes vary with room for major improvement. Thus, this review aims to (1) establish and consolidate knowledge on efficacious eating disorder psychotherapies; (2) highlight select emerging psychotherapeutic interventions; and (3) identify knowledge gaps to better inform future treatment research and development.

**Methods:**

The current review forms part of a series of Rapid Reviews published in a special issue in the Journal of Eating Disorders to inform the development of the Australian-government-funded National Eating Disorder Research and Translation Strategy 2021–2031. Three databases were searched for studies published between 2009 and 2023, published in English, and comprising high-level evidence studies (meta-analyses, systematic reviews, moderately sized randomised controlled studies, moderately sized controlled-cohort studies, and population studies). Data pertaining to psychotherapies for eating disorders were synthesised and outlined in the current paper.

**Results:**

281 studies met inclusion criteria. Behavioural therapies were most commonly studied, with cognitive-behavioural and family-based therapies being the most researched; and thus, having the largest evidence-base for treating anorexia nervosa, bulimia nervosa, and binge eating disorder. Other therapies, such as interpersonal and dialectical behaviour therapies also demonstrated positive treatment outcomes. Emerging evidence supports specific use of Acceptance and Commitment; Integrative Cognitive Affective; Exposure; Mindfulness; and Emotionally-Focused therapies; however further research is needed to determine their efficacy. Similarly, growing support for self-help, group, and computer/internet-based therapeutic modalities was noted. Psychotherapies for avoidant/restrictive food intake disorder; other, and unspecified feeding and eating disorders were lacking evidence.

**Conclusions:**

Currently, clinical practice is largely supported by research indicating that behavioural and cognitive-behavioural psychotherapies are most effective for the treatment of eating disorders. However, the efficacy of psychotherapeutic interventions varies across studies, highlighting the need for investment and expansion of research into enhanced variants and novel psychotherapies to improve illness outcomes. There is also a pressing need for investigation into the whole range of eating disorder presentations and populations, to determine the most effective interventions.

## Abstract (plain language summary)

Evaluating the efficacy of treatment options for eating disorders (EDs) is important and necessary to inform both treatment guidelines and clinical practice. However, treatment outcomes in studies, and in clinical practice, can vary widely. Therefore, this review aimed to pool evidence related to a wide range of psychological treatments to help better understand what gaps in treatment need to be addressed. Using a rapid review method, three academic databases were searched, and 281 articles were identified and analysed. Results indicated that cognitive-behavioural approaches had the most evidence for well-defined EDs (such as anorexia nervosa, bulimia nervosa, and binge eating disorder). However, little research was found on other types of EDs. There was emerging evidence that provided support for self-help, group, and computer/internet-based therapies. Overall, the findings highlighted that more research is required on novel eating disorder treatments beyond what is currently available and being used as ‘gold standard’.

## Introduction

Eating disorders (EDs) are serious mental illnesses with significant psychiatric and medical morbidity and mortality [1]. They are one of the most challenging of the mental illnesses to treat, attributed primarily to their complex biopsychosocial aetiology, ego-syntonic features and resistance to treatment [2]. Psychotherapy, or talk therapy, is a first-line treatment intervention for EDs as it targets psychological and social factors that contribute to disease onset and maintenance [3].

The mechanisms underlying ED symptomatology and diagnoses are multi-factorial, leading to a range of potential therapeutic targets [4]. EDs encompass a broad range of diagnostic presentations requiring different therapeutic foci and, as such, some psychotherapies have been adapted according to specific ED diagnoses in order to provide a more focussed treatment [4]. Independent variables considered critical in the development and maintenance of EDs represent potential therapeutic targets. Psychotherapies target maladaptive behaviours, personality traits and negative affect, which typically span the ED spectrum [4].

Significant advancements to psychotherapeutic interventions for EDs have been made over the past 2 decades, and an extensive evidence base evaluating their efficacy has developed [5]. However, these advances are relative in that they represent some, but not profound, improvements in the effectiveness of available treatments, which may still be improved further. This is particularly important to consider in the context of clinical outcomes, health system resourcing, and research funding which significantly relies on the evidence-base to make informed-decisions and to inform policy.

Results from randomised controlled trials (RCTs) are often used to determine which treatments are recommended in national guidelines. The UK’s National Institute for Clinical Excellence (NICE) [6] guidelines for EDs, consistent with other international guidelines [5, 7], endorse enhanced cognitive-behavioural therapy (CBT-E), Maudsley Anorexia Treatment for Adults (MANTRA) and specialist supportive clinical management (SSCM) for adult Anorexia Nervosa (AN), and family-based therapy (FT-AN) for children and adolescents with AN. For the treatment of adult Bulimia Nervosa (BN), BN-focused guided self-help programs incorporating cognitive-behavioural self-help materials supplemented with brief supportive sessions are recommended. For child and adolescent BN, BN-focused family therapy (FT-BN) is suggested as the primary treatment. For treating Binge Eating Disorder (BED) in adults and children, a BED-focused guided self-help program utilising cognitive-behavioural materials and brief supportive sessions is the recommended approach. For Other Specified Feeding and Eating Disorder (OSFED) and Unspecified Feeding or Eating Disorder (UFED), evidence-based recommendations cannot be made according to NICE guidelines due to a lack of research. In the absence of adequate evidence, NICE suggests a consideration of the presenting symptoms and application of the recommended therapy for the ED it most closely resembles. The guidelines do not provide recommendations for the treatment of Avoidant Restrictive Food Intake Disorder (ARFID).

Despite substantial research into ED aetiology and pathology, few theories have been translated into effective interventions [3]. Considering high rates of relapse among individuals with an ED, and an estimated 50% of cases progressing to a severe and enduring illness, the low efficacy of current treatments is concerning [8]. This is further compounded by the high rates of treatment drop-out commonly observed among patients with an ED [9]. Thus, the current review aims to (1) establish and consolidate the evidence-base of psychotherapies and efficacy for treating eating disorders in order to understand the ED treatment landscape; and (2) identify gaps in research and highlight emerging treatments that warrant further research investment.

## Methods

### Overview and rationale

The Australian Government funded the InsideOut Institute for Eating Disorders (IOI) to develop the Australian Eating Disorders Research and Translation Strategy 2021–2031, [10] in partnership with state and national stakeholders including clinicians, service providers, researchers, and experts by lived experience (including consumers and families/carers). Developed through a 2-year national consultation and collaboration process, the strategy provides the roadmap to establishing EDs as a national research priority and is the first disorder-specific strategy to be developed in consultation with the National Mental Health Commission. To inform the strategy, IOI commissioned Healthcare Management Advisors (HMA) to conduct a series of rapid reviews (RRs) to broadly assess all available peer-reviewed literature on the six DSM-5 listed EDs.

A RR Protocol [11] was utilised to swiftly synthesise evidence in order to guide public policy and decision-making [12]. This approach has been adopted by several leading health organisations including the World Health Organisation [10] and the Canadian Agency for Drugs and Technologies in Health Rapid Response Service [13], to build a strong evidence base in a timely and accelerated manner, without compromising quality. A RR is not designed to be as comprehensive as a systematic review—it is purposive rather than exhaustive and provides actionable evidence to guide health policy [14].

### Search strategy

The RR is a narrative synthesis and follows the PRISMA guidelines [15]. It is divided by topic area and presented as a series of papers. Three research databases were searched: ScienceDirect, PubMed and Ovid/Medline. To establish a wide understanding of the progress made in the field of psychotherapeutic approaches in EDs, the search strategy and eligibility criteria were kept relatively broad.

#### Eligibility criteria

Therefore, included studies were published between 2009 and 2023, in English, and conducted within Western healthcare systems or health systems comparable to Australia in terms of structure and resourcing. The initial search and review process was conducted by three reviewers between 5 December 2019 and 16 January 2020. The search was re-run for dates spanning 16 January 2020 until 28th January 2023 and was conducted by two reviewers.

### Study sampling and included studies

The RR had a translational research focus with the objective of identifying evidence relevant to developing optimal care pathways. Searches therefore used a Population, Intervention, Comparison, Outcome (PICO) approach to identify literature relating to population impact, prevention and early intervention, treatment, and long-term outcomes. Discretionary, purposive sampling predominantly focused on high-level evidence studies such as: meta-analyses; systematic reviews; moderately sized randomised controlled studies (RCTs) (*n* > 50); moderately sized controlled-cohort studies (*n* > 50), and population studies (*n* > 500). However, the diagnoses ARFID and UFED necessitated a less stringent eligibility criterion due to a paucity of published articles. As these diagnoses are newly captured in the DSM-5 (released in 2013, within the allocated search timeframe), the evidence base is emerging and fewer studies have been conducted. Thus, smaller studies (n ≤ 20) and narrative reviews were also considered and included. Grey literature, such as clinical or practice guidelines, protocol papers (without results) and Masters’ theses or dissertations, was excluded. Other sources (which may not be replicable when applying the current methodology) included the personal libraries of authors. This extra step was conducted in line with the PRISMA-S: an extension to the PRISMA Statement for Reporting Literature Searches in Systematic Reviews [16].

**Fig. 1:**
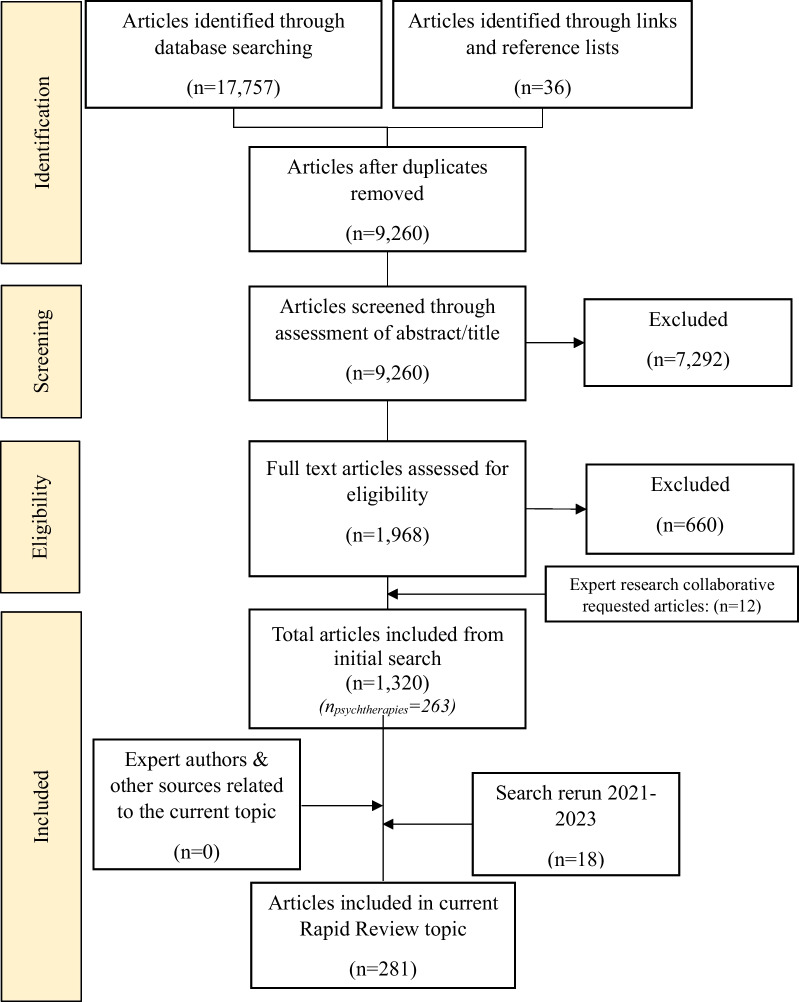
PRISMA diagram—rapid review

Full methodological details including eligibility criteria, search strategy and terms and data analysis are published in a separate protocol paper due to the broad scope of the RR, which included a total of 1320 initial studies [17] (see Fig. 1 for PRISMA flow diagram). Data from included studies relating to psychotherapies were synthesised and are presented in the current review.

## Results

The search identified 281 studies for inclusion in this paper (see Fig. 1 and Table 2). The proceeding section will (1) overview the characteristics of studies found and highlight the most common ED treatment approaches found by the current review; (2) summarise the key findings of the studies; and (3) outline particular psychotherapies targeting specific EDs. Results are presented in the following order: (1) study characteristics and a summary of key findings; (2) overview of common individual-based psychotherapies across a number of eating disorders; (3) an examination of groups-based therapies and (4) technology-based interventions; and finally, (5) an overview of interventions for carers supporting someone living with an ED.

### Study characteristics

Studies focusing on cognitive behavioural therapy (CBT) comprised the largest proportion (30%), followed by family based therapy (FBT) (12%), exposure-based therapies (5%), interpersonal therapy (IPT), dialectical behaviour therapy (DBT), acceptance and commitment therapy (ACT), integrative cognitive affective therapy (ICAT) (all 3%), and other psychotherapies, including mindfulness, emotionally-focused, self-compassion, self-identification, motivational interviewing (MI) and body movement and awareness therapies (2%). Studies investigating self-help, group and technology-based psychotherapy delivery modalities were also included.

Differences in body mass index (BMI), Eating Disorder Examination (EDE) [18], global scores, ED psychopathology, maintenance mechanisms and affect measured pre- and post-treatment were the most commonly measured treatment outcomes. The majority of studies investigated outcomes for the client, who were predominantly female, adult, clinical populations in outpatient settings (Table 1).

**Table 1:** Summary of key findings

Psychotherapy	Key findings
Cognitive behavioural therapy (CBT)	CBT emerged as the primary treatment for individuals with BN and BED. There is consensus regarding its transdiagnostic therapeutic effectiveness across diagnoses given its ability to target illness-maintaining features and reduce binge/purge symptomatology. Strong emerging evidence suggests that CBT may be effective when administered using group therapy, guided self-help and technology-based delivery modalities
Dialectical behavioural therapy (DBT)	Studies focusing on DBT and DBT-BED have indicated that it may be successful at reducing the frequency of binge-eating
Family based therapy (FBT)	FBT should be considered in the first instance for the treatment of children and adolescents with AN (including atypical presentations, OSFED/UFED). It is the most consistently effective treatment for adolescent AN and has been found to be highly cost-effective in an Australian context
Interpersonal therapy (IPT)	IPT has been considered an effective and viable alternative treatment for BN and BED
Technology-based interventions	Emerging evidence is beginning to highlight that technology-based interventions, such as iCBT, CBTR4BN, RecoveryMANTRA, guided computer-based interventions, and virtual-reality may be efficacious treatments for a range of EDS—primarily BED and BN
Guided self-help (GSH)	Research suggests GSH as the recommended first-line treatment for non-underweight EDs, with illnesses characterised by recurrent binge eating, namely BED and BN. Self-help interventions are not recommended in the treatment of AN due to the specialist care required for this disorder
Other therapeutic approaches and treatments for less common eating disorders	Investigations into novel psychotherapeutic approaches are being trialled in RCTs. From the small number of studies included, neither ICAT or ACT demonstrated superior efficacy when compared with an active comparator (CBT), or treatment as usual (TAU) for patients with AN or BN. However, emerging evidence suggests that ICAT may be equally as effective as CBT-E and guided self-help at reducing bulimic tendencies
	Research is very limited for the treatment of ARFID
	Research is very limited in terms of ED carer support

A small proportion of included studies (7%) examined therapeutic outcomes for the carer of someone with an ED and the indirect effect on client outcomes. A full list of included studies is available in Table 2. Results are divided into three sections: (1) psychotherapies, (2) psychotherapeutic delivery modalities, and (3) carers. Within some of the psychotherapies, results are sub-divided into ED presentations AN, BN, BED, ARFID, OSFED and UFED. A summary of key findings can be found in Table 1.

**Table 2:** Included studies table

#	Author, year	Country	N_participants_	N_studies_	Population	Aim	Design	Outcome measure
1	Aardoom et al., 2013 [19]	Worldwide		21	Mixed (mixed cohort, Both sexes)	To review the literature regarding internet-based treatment of EDs	Review (systematic)	ED psychopathology, frequency of binge eating and purging, improving (ED-related) quality of life
2	Accurso et al., 2014 [20]	USA	121		Outpatient (adolescent, both sexes)	To describe change in psychological outcomes for adolescents with AN, and to explore predictors of change	RCT	Severity of eating disorder pathology using the EDE V12.0; depressive symptoms using the BECK Depression Inventory (BDI) and body weight
3	Accurso et al., 2015 [21]	USA	80		Community (Adult, Both sexes)	To examine the temporal relation between therapeutic alliance and outcome in two treatments for BN	RCT	Temporal relation between therapeutic alliance and outcome
4	Agras et al., 2014 [22]	USA	164		Outpatient (adolescent, both sexes)	To compare FBT with systemic family therapy for the treatment of adolescent-onset anorexia nervosa	RCT	Percentage of ideal body weight and remission (≥ 95% of ideal body weight)
5	Agras, Fitzsimmons-Craft and Wilfley, 2017 [23]	N/A		N/A	Mixed (mixed cohort, both sexes)	To describe the evolution of CBT for the treatment of bulimic disorders	Review (Narrative)	Varied
6	Aguera et al., 2013 [24]	Spain	454		Outpatient (adult, females)	To determine differences in treatment response and dropout rates following CBT across the three bulimic-spectrum syndromes	Repeated measures (without follow up)	Full remission defined as total absence of bingeing and purging behaviours and psychological improvement for at least 4 (consecutive)
7	Aguera et al., 2017 [25]	Spain	262		Outpatient (adult, both sexes)	To compare treatment outcomes and clinical predictors between men and women with EDs	Cross-sectional	Clinical predictors of treatment outcome—ED severity, personality and psychopathology
8	Ahmadiankalati, Steins-Loeber and Paslakis, 2020 [26]	N/A		12	Outpatient (mixed cohort, both sexes)	To identify and analyse the current evidence of RCTs to evaluate the effectiveness and acceptability of e-health interventions in the ED field	Review (other)	Acceptability, effectiveness of effect on eating behaviour
9	Alfonsson, Parling and Ghaderi, 2015 [27]	Sweden	96		Inpatient (undefined)	To assess whether behavioural activation is an efficacious treatment for decreasing ED symptoms in patients with obesity and BED	RCT	Frequency of binge-eating; depressive symptoms and mood
10	Anastasiadou et al., 2020 [28]	Spain	106		Outpatient (mixed cohort, both sexes)	To assess the clinical efficacy of a combined mHealth intervention for EDs based on CBT	RCT	ED symptomatology, anxiety, depression and quality of life
11	Anderson et al., 2020 [29]	USA	112		Outpatient (adult, both sexes)	To examine predictors and moderators of two interventions for binge-eating disorder	RCT	Frequency of Objective Binge-eating Episode (OBE) and OBE-abstinence at end-of-treatment (EOT) and 6-month follow up
12	Ariel and Perri, 2016 [30]	USA	572		Outpatient (adult, both sexes)	To evaluate the effects of a behavioural intervention for obesity compared with a nutrition education group on binge eating	RCT	Binge-eating severity using the Binge Eating Scale and weight status, weight status at base line and 6 months and treatment adherence over 6 months
13	Austin et al., 2022 [31]	UK	502		Outpatient (young people, both sexes)	To assess the scalability of First Episode Rapid Early Intervention for ED service model and care pathway	Quasi-experimental (intervention)	Eating Disorder Examination Questionnaire (EDE-Q), Clinical Outcomes in Routine Evaluation, Clinical Impairment Assessment (CIA), The Depression, Anxiety, and Stress Scale—21 items (DASS-21), Work and Social Adjustment Scale (WSAS), Levels of Expressed Emotion Scale (LEE), Psychological Outcome Profiles (PSYCHLOPS), Body mass index
14	Bankoff et al., 2012 [32]	Worldwide		13	Outpatient (mixed cohort, both sexes)	To conduct a literature review to locate referred articles testing dialectical behaviour therapy for the treatment of EDs	Review (systematic)	Treatment efficacy on ED behaviours and other forms of psychopathology
15	Baudinet et al., 2021 [10]	Worldwide		27	Mixed (mixed cohort, both sexes)	To review the quantitative and qualitative evidence-base for multi-family therapy (MFT) for eating disorders regarding change in physical and psychological symptoms, broader individual and family factors, and the experience of treatment	Review (systematic scoping)	Global outcomes, weight, ED psychopathology, and mood
16	Bauer et al., 2012 [33]	Germany	165		Inpatient (adult, females)	To examine the efficacy of a program delivered via the short message service (SMS) and text messaging in support patients after their discharge from inpatient treatment	RCT	Rate of partial remission, 8 months after discharge
17	Beintner, Jacobi and Schmidt, 2014 [34]	Worldwide		73	Outpatient (mixed cohort, both sexes)	To review and analyse participation and outcome in manualized self-help for BN and BED	Systematic review/meta-analysis (combined)	Varied
18	Bentz et al. 2021 [35]	Denmark	157		Outpatient (young people, both sexes)	To evaluate outcomes of FBT for restrictive-type eating disorders, delivered as standard care in a public mental health service	Quasi-experimental (Intervention)	Remission, frequency of hospital admissions and day-patient treatment, frequency of other adaptations within 12 months from commencement of treatment
19	Berking et al. 2022 [36]	Germany	101		Community (adult, both sexes)	To test whether systematically enhancing emotion regulation skills would reduce symptoms of BED	RCT	Binge-eating symptoms, emotion regulation skills
20	Blanchet et al., 2018 [37]	Worldwide		8	Outpatient (adult, both sexes)	To assess the evidence regarding the role of physical activity in BED and to better understand the mechanisms of action	Review (systematic)	Binge-eating episode frequency; changes in other associated comorbidities
21	Boerhaut et al., 2016 [38]	The Netherlands	40		Outpatient (adult, females)	To evaluate the effect of a brief body and movement-oriented intervention on aggression regulation and ED pathology for individuals with ED’s	RCT	Aggression regulation and ED pathology
22	Boerhaut et al., 2017 [39]	The Netherlands	70		Outpatient (adult, both sexes)	To evaluate a body and movement-oriented intervention on aggression regulation, aimed towards reducing anger internalization in patients with an ED	RCT	Aggression regulation
23	Bourion-Bedes et al., 2013	France	108		Inpatient (young people, both sexes)	To determine whether patients’ perception of early therapeutic alliance could predict time to achieve a target weight	Repeated Measure (without follow-up)	Time to achieve a target weight
24	Brauhardt, de Zwaan and Hilbert, 2014 [40]	Worldwide		123?	Community (mixed cohort, both sexes)	To investigate the evidence supporting the impact of therapeutic process aspects on outcomes	Review (systematic)	The impact of therapeutic process aspects on outcome (i.e., process-outcome research)
25	Brewin et al., 2016 [41]	UK	158		Mixed (mixed cohort, both sexes)	To evaluate the effect of a newly developed motivational and psycho-educational guided self-help intervention for people with ED’s on engagement and retention in therapy	Repeated Measures (without Follow Up)	Rates of engagement and completion of therapy
26	Brockmeyer et al., 2019 [42]	Germany	56		Outpatient (adult, both sexes)	To examine if approach bias modification reduces approach-basis automatic action tendencies toward food and may decrease BE and related symptoms	RCT	Number of objective binge-eating episodes (OBEs) as assessed by the Eating Disorder Examination (EDE) and ED symptoms
27	Brockmeyer, Friederich and Schmidt, 2018 [43]	Worldwide		19	Community (adolescent, both sexes)	To synthesise evidence on established and emerging anorexia nervosa treatments and to forecast future development trends	Review (systematic)	Outcomes of clinical trials on established AN treatment
28	Brockmeyer et al., 2021 [44]	Germany	167		Community (adult, both sexes)	To evaluate potential treatment-enhancing effects of CRT on cognitive and clinical outcomes in a large sample of patients with AN in a randomized controlled trial with equal treatment dosage in the comparator condition	RCT	BMI, ED psychopathology, health-related quality of life, motivation to change, set-shifting, central coherence
29	Brown, Mountford, and Waller, 2013 [45]	UK	65		Outpatient (adult, both sexes)	To establish the strength of the therapeutic alliance (TA), to determine if early TA is associated with the completion of CBT and the direction of the relationship between TA and weight gain	Longitudinal (< 5 years)	Weight gain (BMI change), Working Alliance Inventory (WAI-SR), Eating Disorders Examination (EDE-Q)
30	Brownley et al., 2016 [46]	Worldwide		34	Outpatient (adult, both sexes)	To summarise evidence about the benefits and harms of psychological and pharmacologic therapies for adults with BED	Systematic review/meta-analysis (combined)	Abstinence from binge-eating, binge-eating frequency, eating-related psychotherapy symptoms of depression, body weight
31	Butler and Heimberg, 2020 [47]	Worldwide		60	Inpatient and outpatient (mixed cohort, both sexes)	To review the literature on exposure interventions for EDs; exposure and response prevention, in vivo feared food exposure, mirror exposure, FBT with exposure, and virtual reality exposure therapy	Review (systematic)	Testing anxiety levels, calorie intake, BMI
32	Buerger et al., 2021 [48]	Worldwide	487	19	Mixed (young people, both sexes)	To estimate the efficacy of third-wave interventions to reduce ED symptoms in adolescents in randomized controlled trials (RCTs) and uncontrolled studies	Systematic review/meta-analysis (combined)	ED psychopathology (EDE, EDEQ, Eating Disorder Inventory-2, Eating Disorder Inventory-3, Structured Interview for Anorexic and Bulimic Disorders for DSM-IV and ICD-10)
33	Byrne et al., 2011 [49]	Australia	125		Community (adult, both sexes)	To examine the effectiveness of Enhanced Cognitive Behaviour Therapy (CBT-E) for EDs in an open trial for adults with the full range of EDs found in the community	Quasi-experimental (intervention)	Categorical measures of recovery, dimensional measures of change in the severity of ED features, compensatory behaviours, dietary restraint, eating, weight and shape concerns, measures of additional variables specified in the transdiagnostic model, and measures of other associated psychopathology
34	Calugi et al. 2021 [50]	Italy	214		Inpatient (mixed cohort, both sexes)	To compare the change in eating-disorder feature networks in patients with anorexia nervosa after treatment with intensive enhanced cognitive behaviour therapy (CBT-E)	Quasi-experimental (intervention)	BMI, EDEQ
35	Cardi et al., 2020 [51]	UK	187		Outpatient (adult, both sexes)	To examine if adding a brief online intervention focused on enhancing motivation to change and the development of a recovery identity would improve outcomes in adults with AN	RCT	Body Mass Index, frequency of eating disorder symptoms, psychological wellbeing and work and special adjustment and anxiety
36	Carrard et al., 2011 [52]	Switzerland	127		Community (adult, females)	To evaluate the use of an online guided self-treatment programme for BN and to determine predictors of outcome	Quasi-experimental (intervention)	Eating Disorders Inventory-2 (EDI-2) and The Symptom Checklist-revised (SCL-90R)
37	Carrard et al., 2011 [53]	Switzerland	74		Community (adult, females)	To evaluate the efficacy of an internet guided self-help treatment programme, based on CBT, for adults with threshold and subthreshold BED	RCT	Number of objective binge-eating episodes (OBEs) as assessed by the Eating Disorder Examination (EDE-Q) and ED symptoms score (EDI-2), perceived hunger, psychological health, depression, self-esteem and quality of life
38	Catalan-Matamoros et al., 2011 [54]	Spain	28		Outpatient (adult, both sexes)	To analyse the feasibility of Basic Body Awareness Therapy in people with EDs	RCT	Eating Disorder Inventory, Eating Attitude Test, Body Attitude Test and Quality of Life Scale (SF-36)
39	Cesa et al., 2013 [55]	Italy	90		Outpatient/inpatient (adult, females)	To test the brief and long-term clinical efficacy of an enhanced CBT in morbidly obese patients with BE disorders compared with standard CBT	RCT	Weight loss, weight loss maintenance, BED remission, body-satisfaction improvement
40	Chami et al., 2022 [56]	UK	78		Community (adult, both sexes)	To examine the feasibility, acceptability, and effect sizes of clinical outcomes of an intervention that combines inhibitory control training (ICT) and implementation intentions (if–then planning) to target binge eating and eating disorder psychopathology	RCT	ED psychopathology (EDE-Q), weight, self-regulation of eating, food valuation, food approach, depression and anxiety
41	Chang, Delgadillo and Waller, 2021 [57]	Worldwide		26	Outpatient (mixed cohort, both sexes)	To examine literature that states that early response is a well-established predictor of positive outcomes at the end of psychological treatments for common mental disorders	Systematic Review/Meta-Analysis (Combined)	The statistical significance and magnitude of the association between early response to ED treatment and outcomes
42	Chen et al., 2017 [58]	USA	109		Outpatient (adult, females)	To improve the outcome of clients with weak initial response to guided self-help CBT	RCT	Frequency of and abstinence from ED behaviour and psychopathology using the EDE
43	Ciao et al., 2015 [59]	USA	80		Outpatient (adolescent, both sexes)	To examine predictors of psychological change among adolescents with BN comparing FBT to supportive psychotherapy	RCT	Abstinence from bingeing and purging; psychological outcomes (cognitive ED pathology (i.e., Shape/Weight Concerns, Eating Concerns, and Restraint), depression, and self-esteem
44	Clyne et al., 2010 [60]	New Zealand	23		Outpatient (adult, females)	To examine the efficacy of a treatment for BED designed to increase recognition and regulation of negative emotion, replicating and extending a previous investigation	Repeated measures (with follow up)	Binge-abstinence rates, pre and post study treatment effects
45	Compare and Tasca, 2016 [61]	Italy	118		Outpatient (adult, both sexes)	To investigate the change and relationship between BE episodes and weight across focused group therapy and combined therapy of EFGT plus dietary counselling for BED	Longitudinal (< 5 years)	Binge-eating episodes and body weight
46	Compare et al., 2013 [62]	Italy	189		Outpatient (adult, both sexes)	To test the effect on psychopathology and QOL of Emotionally Focused Therapy, Dietary Counselling, and Combined Treatment in patients with BED and obesity	Longitudinal (< 5 years)	Health-related quality of life, using ORWELL-97; attitudes toward eating (Eating Inventory—EI), binge eating (Binge Eating Scale—BES) and body uneasiness (Body Uneasiness Test—BUT) was performed at baseline, end-of-treatment, and 6-month follow-up
47	Coomber and King, 2012 [63]	Australia	56		Community (mixed cohort, both sexes)	To examine predictors of both carer burden and carer psychological distress in ED carers	Cross-sectional	Eating Disorders Symptom Impact Scale (EDSIS), Carers’ Needs Assessment Measure (CaNAM), General Health Questionnaire-12 (GHQ-12), Brief COPE, Social Support Questionnaire (SSQ6), Family Questionnaire (FQ)
48	Coomber and King, 2013 [64]	Australia	42		Community (adult, both sexes)	To conduct a preliminary longitudinal examination of the predictors of carer burden and psychological distress for carers of those with an ED	Longitudinal (< 5 years)	Eating Disorders Symptom Impact Scale (EDSIS), Carers’ Needs Assessment Measure (CaNAM), General Health Questionnaire-12 (GHQ-12), Brief COPE, Social Support Questionnaire-Short Form (SSQ6), Family Questionnaire (FQ)
49	Costa and Melnik, 2016 [65]	Worldwide		N/A	Community (mixed cohort, both sexes)	To compile findings of relevant scientific papers, such as randomized controlled trials, systematic reviews, meta-analysis, guidelines and narrative reviews of literature, in order to promote knowledge about effectiveness of psychosocial interventions in EDs along time, in addition to showing the need for further research in specific areas	Review (narrative)	Symptomatic remission; for AN- BMI; for BN-100% withdrawal from binge eating, bulimic symptoms; for BED/eating disorder not otherwise specified (EDNOS)-remission of bulimic symptoms, weight BMI
50	Courbasson, Nishikawa and Shapira, 2010 [66]	Canada	38		Mixed (mixed cohort, both sexes)	To examine treatment outcome for individuals with BED and those who often evidenced comorbid substance use disorders	Repeated measures (without follow up)	Measures of objective binge-eating episodes; disordered eating attitudes; alcohol and drug addiction severity; depression
51	Couturier, Kimber and Szatmari, 2012 [67]	Worldwide		6	Outpatient (adolescent, both sexes)	To review and evaluate the efficacy of FBT compared with individual treatment among adolescents with EDs	Systematic review/meta-analysis (combined)	Remission rate at end of treatment, 6 and 12 month follow up
52	Craig et al., 2019 [68]	UK	54		Community (adolescent, both sexes)	To consider the effectiveness of CBT for EDs where family-based treatment was either not fully effective or not applicable	Case series	Eating attitudes and clinical impairment, and weight change
53	Dakanalis et al., 2014 [69]	Italy	679		Outpatient (adult, both sexes)	To evaluate and compare the original cognitive-behavioural model and the enhanced cognitive-behavioural model	Cross-sectional (correlational)	Diagnosis of ED
54	Dalle Grave et al., 2015 [70]	Worldwide		68	Outpatient (adolescent, both sexes)	To evaluate the effects of CBT on non-underweight adolescents with an ED	Review (systematic)	Body weight and BMI; Eating Disorder features using EDE-Q6.0; general psychiatric features from Global Severity Index (GSI)
55	Daniel, Poulsen and Lunn, 2016 [71]	Denmark	70		Community (adult, females)	To perform secondary analyses on the relation between attachment and pre-treatment symptom levels	Repeated measure (with follow-up)	Attachment and pre-treatment symptom levels, pre-treatment attachment treatment outcome moderation, change in client attachment associated with symptomatic change, changes to client attachment
56	Daniel, Lunn and Poulsen, 2015 [72]	Denmark	70		Outpatient (adult, both sexes)	To perform analyses of the relation between attachment and pre-treatment symptom levels for BN with respect to outcome, symptoms and client attachment	RCT	Moderation/change in assessment at intake included Eating Disorder Examination (EDE), Adult Attachment Interview, Symptom Checklist 90-R
57	de Jong, Schoorl and Hoek, 2018 [73]	Worldwide		7	Outpatient (adult, both sexes)	To provide an update of CBT-E effectiveness studies on BN, BED and transdiagnostic samples	Review (systematic)	Post treatment remission rates
58	de Zwaan et al., 2017 [74]	Germany	178		Outpatient (adult, both sexes)	To evaluate the efficacy of internet-based guided self-help compared with traditional, individual face-to-face CBT	RCT	Difference in number of days with objective binge-eating episodes (OBEs) during the previous 28-days between baseline and treatment; OBEs at follow ups, ED and general psychopathologic findings, body mass index, and quality of life
59	Diaz-Ferrer et al., 2015 [75]	Spain	29		Outpatient (adult, females)	To compare the efficacy of two body exposure techniques through psychological and neuroendocrine indices within and between successive sessions	RCT	Body satisfaction and changes in subjective discomfort
60	Dolemeyer et al., 2013 [76]	Worldwide		8	Mixed (mixed cohort, both sexes)	To evaluate the efficacy of internet-based interventions for the treatment of different EDs in adults	Review (systematic)	Varied
61	Dray and Wade, 2012 [77]	Worldwide		9	Community (mixed cohort, both sexes)	To examine the utility of the transtheoretical model and the efficacy of motivational interviewing in predicting outcome of research in EDs	Review (critical)	Utility of the transtheoretical model in predicting outcome, and efficacy of motivational interviewing
62	Egger et al., 2016 [78]	Germany	156		Outpatient (adult, females)	To determine the cost-effectiveness of outpatient focal psychodynamic psychotherapy, CBT-E, and optimized TAU in adult women with AN	RCT	Cost effectiveness
63	Eisler et al., 2016 [79]	UK	169		Outpatient (adolescent, both sexes)	To compare two outpatient ED focussed family interventions; multi-family therapy and single family therapy	RCT	Achieving good or intermediate outcome on the Morgan-Russell scales at the end of treatment
64	Ertelt et al., 2011 [80]	N/A		116?	Outpatient (adult, females)	To examine ratings of therapeutic alliance factors in telemedicine and face-to-face CBT	Review (narrative)	Working Alliance Inventory (WAI) completed by patients and therapists at weeks 2, 8, and 16
65	Fairburn et al., 2009 [18]	UK	154		Outpatient (adult, both sexes)	To compare two cognitive-behavioural treatments for outpatients with EDs	RCT	ED features and mood intolerance, clinical perfectionism, low self-esteem, or interpersonal difficulties
66	Fairburn et al., 2013 [81]	UK	99		Outpatient (adult, both sexes)	To establish the immediate and longer-term outcome following CBT-E	Repeated Measure (with follow-up)	BMI, ED features and psychiatric features
67	Fairburn et al., 2015 [82]	UK	130		Outpatient (adult, both sexes)	To compare CBT with interpersonal psychotherapy	RCT	ED features and remission rate using the Eating Disorder Examination Interview (EDE and EDE-Q6.0)
68	Fernandez-Aranda et al., 2015 [83]	Spain	38		N/A	To compare outcomes of outpatient CBT + serious video game (SVG), with outpatient CBT—SVG, and effect in reducing emotional expression and levels of anxiety than CBT—SVG	Control Trial	Drop-out, partial remission, and total remission
69	Ferrer-Garcia and Gutierrez-Maldonado, 2012 [84]	N/A		N/A	Mixed (Mixed cohort, both sexes)	To review research of virtual reality in the study, assessment, and treatment of body image disturbances in EDs and nonclinical samples	Review (narrative)	N/A
70	Ferrer-Garcia et al., 2019 [85]	N/A		58	Outpatient (adult, both sexes)	To assess the 6-month follow up of virtual reality cue exposure therapy as a second-level treatment for BN patients and binge ED following CBT	Review (narrative)	Frequency of binge-eating episodes and purging using the ED Examination Interview 12.0D
71	Ferrer-Garcia et al., 2017 [86]	Spain	64		Outpatient (adult, both sexes)	To assess virtual reality cue exposure therapy as a second-level treatment for BN patients and binge eating disorder following CBT	RCT	Frequency of binge-eating episodes and purging using the ED Examination Interview 12.0D
72	Fioravanti et al., 2014 [87]	Italy	102		Community (adult, females)	To evaluate whether the emotional eating profile of ED patients changes over time and the effects of psychotherapeutic intervention	Repeated Measure (without follow-up)	Emotional eating
73	Fisher et al., 2019 [88]	Australia		25	Outpatient (mixed cohort, both sexes)	To evaluate the efficacy of family therapy approaches compared with standard treatment and other treatments for AN	Review (other)	Remission post intervention, remission long-term follow up, and mortality at long-term follow up
74	Fitzsimmons-Craft et al., 2020 [89]	USA	690		Outpatient (adult, females)	To determine whether a coached, digital, CBT intervention improves outcomes for college women with EDs compared with referral to usual care	RCT (cluster)	Changes in overall ED psychopathology, binge eating and compensatory behaviours, ED behaviour frequencies, depression, anxiety, clinical impairment, academic impairment and realised treatment access
75	Fitzsimmons-Craft et al., 2023 [90]	USA	90		Mixed (adult, females)	To estimate the preliminary feasibility and effectiveness of a CBT-based mobile intervention plus treatment as usual (TAU), offered with and without an accompanying social networking feature	RCT	Feasibility, ED psychopathology, frequency of ED behaviours, anxiety, depression and suicidal ideation, clinical impairment, BMI, dietary restraint, weight/shape concern, AN Stages of Change Questionnaire, social support, duration of illness
76	Fogarty, Ramjan and Hay, 2016 [91]	Worldwide		4	N/A	To explore the benefits, effects and experiences of mentoring on those with an ED or disordered eating	Systematic review/meta-analysis (combined)	Varied
77	Folke, Daniel and Poulsen, Lunn 2016 [92]	Denmark	70		Community (adult, both sexes)	To investigate the relation between clients’ attachment patterns and the therapeutic alliance in two psychotherapies for BN	RCT	Client attachment patterns and therapeutic alliance
78	Fox, Dean and Whittlesea, 2015 [93]	Worldwide		20	Mixed (mixed cohort, both sexes)	To establish the acceptability, feasibility and approximate size of the effect of adding a carer intervention to treatment as usual for adolescents with AN	Systematic review/meta-analysis (combined)	Impact upon family members, cognitive appraisals and caregiving experience
79	Gan et al., 2021 [94]	Worldwide		19	Mixed (mixed cohort, both sexes)	To synthesize the best available evidence regarding the effectiveness of non-pharmacological interventions on body mass index (BMI), body dissatisfaction, depression and anxiety among individuals with anorexia nervosa (AN)	Systematic review/meta-analysis (combined)	BMI, body dissatisfaction, depression, anxiety
80	Gale, Gilbert and Goss, 2014 [95]	UK	99		Outpatient (adult, both sexes)	To evaluate the principle that compassion focused therapy can be used with people with EDs and can improve symptomatology	Repeated measure (without follow-up)	Compassion focused therapy outcomes for people with EDs
81	Gallagher et al., 2014 [96]	Canada	102		Outpatient (adults, females)	To conceptualise interpersonal learning as the convergence over time between an individual’s and the group’s perception of the individual’s cohesion to the group	Repeated measures (without follow up)	Cohesion questionnaire—individual version (CQ-1) score and Cohesion to the Group (CQ-G) rating, administered every fourth-group session
82	Garrett et al., 2014 [97]	USA	21		Outpatient (adult, females)	To investigate brain activation and set-shifting and central coherence tasks in patients with AN	Repeated measure (with follow-up)	Central coherence and set shifting
83	Godart et al., 2022 [98]	France	60		(Young people, females)	To present the results of a 54-month post-randomization follow-up of a previously reported randomized controlled trial that compared two post-hospitalization outpatient treatment programs: Treatment As Usual alone versus Systemic Family Therapy added to Treatment As Usual	RCT	Morgan and Russell global outcome categories, Global Outcome Assessment Schedule score, BMI, amenorrhea, number of hospitalisations, ED symptoms
84	Gomez-Castillo et al., 2018 [99]	Spain	348		Outpatient (mixed cohort, both sexes)	To examine the differences in ED symptoms in parents and their children as patients with AN, bulimia nervosa, unspecified ED and a control group	Case series	ED symptoms as measured on the Eating Disorder Inventory (EDI)
85	Gorrell, Loeb and Le Grange, 2019 [100]	N/A		N/A	Outpatient (adolescents, both sexes)	To describe the role of family engagement within FBT of EDs and the interventions	Review (narrative)	Not defined
86	Graham and Walton, 2011 [101]	UK	40		Outpatient (adult, both sexes)	To offer CD-Rom CBT self-help treatment, in a locality-based outpatient NHS ED Service to patients who have BE disorder and BN	Cross-sectional	Wellbeing and functioning, problems and risk; Bulimic Sub Scale of the Eating Disorder Index; dropout rate
87	Gregertsen et al., 2019 [102]	Worldwide		27	Outpatient (mixed cohort, both sexes)	To summarise the evidence base examining baseline predictors of drop-out and outcome in anorexia nervosa treatment	Systematic review/meta-analysis (combined)	Baseline predictors of drop-out rate
88	Grenon et al., 2017 [103]	Canada		27	Outpatient (adults, both sexes)	To review the effect of group psychotherapy compared to both wait-list controls and other active treatments for adults with ED’s	Meta-analysis	Abstinence rates of binge-eating and/or purging; ED psychopathology
89	Griffen, Naumann and Hilderbrant, 2018 [104]	N/A		N/A	Outpatient (mixed cohort, both sexes)	To discuss how individuals respond when looking in a mirror and the use of mirrors therapeutically; to evaluate the benefits, clinical indications and technical considerations for the use of mirror exposure therapy	Review (narrative)	Varied
90	Griffiths et al., 2015 [105]	Australia	317		Community (mixed cohort, both sexes)	To examine the prevalence and correlates of stigma as reported by individuals with EDs	Cross-sectional	Stigma Scale, Self-Stigma of Seeking Help scale (SSOSH), Depression Anxiety Stress Scales (DASS-21), Self-Esteem Scale (SES), Eating Disorder Examination Questionnaire (EDE-Q)
91	Griffiths et al., 2018 [106]	Australia	425		Outpatient (adults, both sexes)	To quantitatively examine individuals’ attitudes towards accessing treatment and perceived barriers to seeking treatment for EDs	Case series	Treatment attitudes, treatment barriers, and eating disorder symptom severity
92	Grilo et al., 2011 [107]	USA	125		Outpatient (adults, both sexes)	To compare CBT, Behaviour Weight Loss (BWL), and sequential approach in which CBT is delivered first followed by BWL (CBT + BWL)	RCT	Binge-eating remission rates and BMI losses
93	Grilo et al., 2013 [108]	USA	48		Outpatient (mixed cohort, both sexes)	To examine the effectiveness of a self-help treatment as a first line primary care intervention for BED in obese patients	RCT	Rates of remission of binge-eating; frequency of objective bulimic episodes using the Eating Disorder Examination Interview (EDE); depression using BDI
94	Grilo et al., 2020 [109]	USA	191		Community (adult, both sexes)	To examine longer-term effects of behavioural weight loss and Stepped Care for BED and obesity through 12-month follow-up after completing treatments	RCT	Frequency of Binge-eating, body weight
95	Groff, 2015 [110]	N/A		6	Community (adult, both sexes)	To review the current empirical research regarding CBT-E in the treatment of EDs	Review (other)	Effectiveness of enhanced cognitive behavioural therapy (CBT-E)
96	Grover et al., 2011 [111]	UK	64		Community (mixed cohort, both sexes)	To evaluate the efficacy of a novel web-based systemic cognitive-behavioural intervention for carers of people with AN	RCT	Depression and anxiety; expressed emotion (EE)
97	Grover et al., 2011 [112]	UK		27	Community (adult, both sexes)	To evaluate the feasibility and acceptability of a novel systemic CBT intervention for carers of people with anorexia nervosa (AN)	Review (other)	Hospital Anxiety and Depression Scale (HADS), Experience of Care Giving Inventory (ECI), The Eating Disorder Symptom Impact Scale (EDSIS)
98	Hay and Claudino, 2015 [113]	Worldwide		8	Mixed (mixed cohort, both sexes)	To conduct a review on the effects of online interventions for people with BN	Review (systematic)	Varied
99	Hay et al., 2009 [114]	Worldwide	3054	48	Outpatient (adult, both sexes)	To evaluate the efficacy of CBT, CBT-BN and other psychotherapies	Review (systematic)	Efficacy of CBT, CBT‐BN and other psychotherapies
100	Hay et al., 2022 [115]	Australia	98		Outpatient (adults, both sexes)	To explore the efficacy of a novel intervention integrating Cognitive Behavioural Therapy- Enhanced (CBT-E) and weight management for people with recurrent binge eating episodes and high BMI with respect to physical, psychopathological and quality of life outcomes	RCT	Metabolic parameters, health-related quality of life, general psychological and ED symptoms, ED diagnostic status outcomes
101	Hay, Touyz, and Sud, 2012 [116]	Worldwide		N/A?	Mixed (mixed cohort, both sexes)	To conduct a review of RCTs of treatment for chronic AN participants and identify research informing novel therapeutic approaches	Review (systematic)	Efficacy of treatment
102	Haynos et al., 2017 [117]	USA	80		Outpatient (adult, both sexes)	To use statistical analyses to identify ED subtypes within individuals with BN and to predict clinical outcomes	RCT	Dimensional Assessment of Personality Pathology-Basic Questionnaire (DAPP-BQ), Eating Disorder Examination (EDE)
103	Hazzard et al., 2021 [118]	USA	112		Community (adults, both sexes)	To examine childhood abuse and post‐traumatic stress disorder (PTSD) as predictors and moderators of binge‐eating disorder (BED) treatment outcomes in a randomized controlled trial comparing Integrative Cognitive‐Affective Therapy with cognitive‐behavioural therapy administered using guided self‐help	RCT	Binge-eating frequency, EDE
104	Herbrich-Bowe et al., 2022 [119]	Germany	56		Inpatient (young people, females)	To compare CRT with non‐specific cognitive training (NSCT) on set‐shifting and central coherence ability as well as self‐reported everyday life flexibility in the first RCT in adolescent inpatients with AN	RCT	Cognitive flexibility, central coherence performance
105	Herzog et al., 2022 [120]	Germany	247		Outpatient (adult, females)	To evaluate the long-term outcomes of a well described and fairly homogeneous sample of adult patients with anorexia nervosa and, to examine whether the treatment advantages found at the 1-year follow-up would persist more than 4 years later	Longitudinal	BMI, eating pathology, mental health outcomes
106	Hibbs et al., 2015 [121]	UK		13	Community (mixed cohort, both sexes)	To perform a meta-analysis of quantitative studies that have described the impact of interventions on caregivers	Meta-analysis	Level of carer distress and carer burden; expressed emotion
107	Hilbert et al., 2015 [122]	USA	205		Outpatient (adults, both sexes)	To analyse effects of rapid response across different treatments for BE disorder	RCT	Rates of remission from binge-eating, Global Eating Disorder Psychotherapy Pathology at posttreatment, 6, 12,18 and 24-month follow ups
108	Hilbert et al., 2019 [123]	Worldwide	7515	81	Community (adult, both sexes)	To provide a meta-analysis on the efficacy of psychological and medical treatments for BED	Meta-analysis	Binge-eating episodes and abstinence from binge eating plus secondary outcomes; ED psychopathology and body image, and general psychopathology
109	Hill, Craighead and Safer, 2011 [124]	USA	32		Outpatient (adult, females)	To investigate a modified version of DBT for BN, entitled appetite focused DBT	RCT	Rate of response to treatment and number of BN symptoms
110	Hodsoll et al., 2017 [125]	UK	149		Outpatients (adolescents, both sexes)	To examine the impact of Collaborative Care Skill Training Workshops on carers’ coping strategies etc., as well as unexplored dimension of accommodating and enabling of their loved one's ED behaviour	RCT	Level of Carer skills; accommodation and enabling behaviour; time spent care giving; body mass index; frequency of admissions
111	Högdahl, Birgagard and Bjorck, 2013 [126]	Sweden	79		Outpatient/inpatient (adults, both sexes)	To investigate the effects of a bibliotherapy-based CBT-based guided self-help with internet support in a clinical setting	Repeated measures (without follow up)	Pre and post treatment symptoms measured by the Eating Disorder Questionnaire (EDE-Q) and the Eating Disorder Inventory 2
112	Högdahl et al., 2023 [127]	Sweden	150		Outpatient (adults, both sexes)	To evaluate effects of two types of internet-based cognitive behavioural therapy and a structured day patient program, the latter being a standard treatment at an eating disorder clinic at the time for the study	RCT	ED pathology, self-image, clinical impairment
113	Hoyle et al., 2013 [128]	Australia	37		Community (Mixed Cohort, Both sexes)	To perform a meta-analysis of quantitative studies that have described the impact of interventions on caregivers	RCT	Carer distress, high-expressed emotion (EE), and care-giving burden associated with ED symptoms
114	Hughes et al., 2014	Australia	N/A		Outpatient (adolescents, both sexes)	To describe the change experienced within a multidisciplinary specialist ED service when a new model of care was implemented	Case study	Admission rate, re-admission rate, number of total bed days, completion of treatment, program success
115	Hughes et al., 2018 [129]	Australia	198		Outpatient (adolescents, both sexes)	To examine attendance patterns of families in FBT and the impact on outcome	Case series	Weight and ED symptoms at end of treatment
116	Hughes et al., 2017 [130]	Australia	42		Outpatient/inpatient (adolescents, both sexes)	To examine engagement in and outcomes of FBT for adolescents with atypical anorexia nervosa	Case series	Engagement in FBT (i.e., length and dose of treatment), reduction in ED symptoms as measured by the EDE, reduction in psychological symptoms (i.e., CDI, RSE, YBC-EDS, and CYBOCS), absence of binge eating and purging, return of menses and change in weight
117	Jansingh et al., 2020 [5]	N/A		3	Outpatient (mixed cohort, both sexes)	To overview the recent literature on psychological treatment for young adults/adults with AN and discuss the implications of the findings for clinical practice	Review (narrative)	Available treatment outcomes and their effectiveness
118	Jenkins et al., 2021 [131]	UK	180		Outpatient (adults, both sexes)	To investigate the effectiveness and cost-effectiveness of guided self-help via face-to-face meetings and a more scalable method, providing support via email	RCT	Overall severity of eating psychopathology and for cost-effectiveness, binge-free using symptom abstinence
119	Jenkins, Morgan and Houlihan, 2019 [132]	UK	63		Outpatient (adult, both sexes)	To investigate the effectiveness of CBT for EDs in a 'real-world' setting	Quasi-experimental (intervention)	Height and weight, and remission
120	Jewell et al., 2016 [133]	Worldwide		13	Outpatient (mixed cohort, both sexes)	To critically review the evidence for ED-focused family therapy using a modern paradigm	Review (critical)	Varied
121	Jones and Clausen, 2013 [134]	Denmark	205		Outpatient (mixed cohort, females)	To evaluate the efficacy of a brief group CBT program in treating a large cohort of patients diagnosed with BN	Repeated measures (without follow up)	Bulimia-related behavioural symptoms and bulimia-related distress using EDE
122	Jones et al., 2012 [135]	UK	48		Outpatient (adults, both sexes)	To investigate outcomes of a Guided Self-Help (GSH) programme for BE; and to compare profiles of treatment completers and non-completers; and to qualitatively explore reasons for non-completion	Case study	Gender, age, height and weight; Eating Disorder Examination (EDE), Eating Disorder Examination-Questionnaire (EDE-Q), Beck Depression Inventory-2 (BDI), Work and Social Adjustment Scale (WSAS)
123	Juarascio et al., 2013 [136]	USA	140		Inpatient (adult, females)	To examine the efficacy of an Acceptance and Commitment Therapy (ACT) based group treatment for ED's and to see if the addition of ACT residential groups to TAU would improve treatment outcomes	RCT	Disordered eating using the Eating Disorder Examination Questionnaire (EDE-Q)
124	Juarascio et al., 2022 [137]	USA	56		Community (adults, all sexes)	To assess the feasibility and acceptability of the CBT + system when used in conjunction with CBT, evaluate the ability of the Just-in-time, adaptive interventions (JITAIs) within the CBT + system to improve skill utilization, examine pre- to post-treatment changes in ED symptoms among individuals receiving JITAIs alongside CBT+, and provide a preliminary estimate of the independent efficacy of JITAIs within the CBT + system to inform design of a future fully powered randomized controlled trial	Quasi-experimental (intervention)	Eating pathology, participant skill use, app usage, acceptability
125	Juarascio et al., 2023 [138]	USA	59			To test a novel treatment approach for binge eating targeting reward imbalance, called Reward Re-Training (RRT), in comparison to a therapeutic attention control condition (supportive psychotherapy)	RCT	ED symptoms, depressive symptoms, alcohol and substance use, quality of life
126	Juarascio, Forman and Herbert, 2010 [139]	USA	55		Outpatient (adults, both sexes)	To examine several questions related to the treatment of eating pathology within the context of a larger RCT that compared standard CBT	RCT	The severity of depressive symptoms (BDI-II), anxiety (BAI), Eating Pathology (EPI), life satisfaction (QOLI), level of functioning (GAF, APA, 2000)
127	Kaidesoja, Cooper and Fordham, 2022 [140]	Worldwide		44	Mixed (adults, both sexes)	To map and examine the systematic review evidence base regarding the effects of cognitive-behavioural therapy (CBT) for eating disorders (EDs), especially against active interventions	Review (systematic)	ED behaviours and psychopathology
128	Kambanis and Thomas, 2023 [141]	Worldwide		N/A	Mixed (mixed cohorts, both sexes)	To review the literature pertaining to the assessment and treatment of avoidant/restrictive food intake disorder (ARFID) 10 years following its introduction to DSM-5	Review (narrative)	Psychological evaluation, medical evaluation, treatment, comorbidities, family-based approach, comorbidities, other treatment approaches
129	Karekla, Nikolaou and Merwin, 2022 [142]	USA	92		Community (young people, females)	To evaluate an innovative digital gamified Acceptance and Commitment early-intervention program (AcceptME) for young females showing signs and symptoms of an ED and at high risk for an ED	RCT	Weight Concern Scale, Eating Disorder Diagnostic Scale, EDE-Q, quality of life, body dissatisfaction and feelings of being fat, body-image related thoughts and feelings, Body Image Avoidance Questionnaire
130	Katterman et al., 2014 [143]	Worldwide		14	Outpatient (adults, both sexes)	To review 14 studies that investigated mindfulness meditation as the primary intervention and assessed binge eating, emotional eating, and/or weight change	Review (systematic)	Binge-eating, emotional-eating and/or weight change
131	Katzman et al., 2010 [144]	USA	225		Outpatient (adult, both sexes)	To conduct a RCT in the treatment of BN, comparing CBT versus motivational enhancement and followed by group versus individual CBT	RCT	Patient improvement
132	Keegan, Tchanturia and Wade, 2021 [145]	Worldwide		63	Mixed (adult, both sexes)	To compare previously documented inefficiencies in central coherence and set-shifting between people with non-underweight EDs and people with AN	Systematic review/meta-analysis (combined)	Central coherence, set-shifting
133	Kelly and Carter, 2015 [146]	Canada	41		Outpatient (adults, both sexes)	To compare a compassion-focused therapy-based self-help intervention for BED to a behaviourally based intervention	RCT	Body-Mass Index, EDE-Q, Binge Eating Frequency, Self-Compassion Scale
134	Klein, Skinner and Hawley, 2013 [147]	USA	N/A		Outpatient (adults, females)	To examine two condensed adaptations of dialectical behaviour therapy for BE	RCT	Core binge-eating symptoms and bulimic symptoms, interoceptive awareness
135	Knott et al., 2015 [148]	UK	272		Outpatient (adult, both sexes)	To evaluate treatment outcomes with CBT for adults with bulimia and to compare this with a previously published RCT	Case series	Measurement of ED psychotherapy using EDE-Q scores
136	Konig et al., 2018 [149]	Germany	147		Outpatient (mixed cohort, both sexes)	To determine the cost-effectiveness of individual face-to-face CBT compared to therapist guided Internet-based self-help in overweight or obese adults with BED	RCT	Long-term effectiveness (measured in Binge-free days) as well as the development of costs beyond the end of treatment, over a 22-month observation period
137	Kroger et al., 2010 [150]	Germany	24		Inpatient (adult, females)	To determine if adapted dialectical behaviour therapy for borderline personality disorder and EDs might improve disorder related complaints	Repeated measures (with follow up)	Remission rate, mean body weight
138	Lammers et al., 2022 [151]	Netherlands	175		Community (adults, both sexes)	To evaluate whether the results of a quasi-randomized study, comparing dialectical behavior therapy for binge-eating disorder (DBT-BED) and an intensive, outpatient cognitive behaviour therapy (CBT +) in individuals with BED, would be replicated in a nonrandomized study with patients who more closely resemble everyday clinical practice	Quasi-experimental (intervention)	ED pathology, emotion regulation, general psychopathology
139	Lampard, 2011 [152]	Australia	162		Outpatient (adult, females)	To compare and evaluate the original and the enhanced cognitive-behavioural models of BN using structural equation modelling	Cross-sectional	Variables outlined in the original CB-BN model, self-esteem, perfectionism, mood intolerance, interpersonal problem
140	Lane-Loney et al., 2022 [153]	USA	81		Inpatient (adolescents, both sexes)	To describe our protocol for treating ARFID at our PHP, to provide case studies exemplifying how our treatment protocol is modified for each unique ARFID presentation, and to explore the relative effectiveness of our treatment program for three different ARFID presentations		Anthropometrics, food acceptance/fear survey, anxiety symptoms, depressive symptoms,
141	Lavender et al., 2012 [154]	UK	74		Outpatient (adults, both sexes)	To test a novel treatment based on a hypothesis that a focus on broader emotional and social/interpersonal issues underlying ED's would increase treatment efficacy, on an Emotional and Social Mind Training Group, against a CBT Group treatment	RCT	EDE global score
142	Lazaro et al., 2011 [155]	Spain	160		Outpatients (adolescents, both sexes)	To evaluate self-esteem and social skills in adolescent ED patients before/after specific group therapy in a Day Hospital Programme	Case study	The Piers–Harris Children’s Self-Concept Scale (PHC-SCS), the Self-Esteem in Eating Disorders Questionnaire (SEED) and the Socialization Battery (BAS-3), at the beginning of group therapy, and after the completion of 8-sessions
143	Le et al., 2017 [156]	Australia	N/A		Community (adult, both sexes)	To model the cost-effectiveness of specialist-delivered CBT for BN compared to no intervention	Modelling (statistical)	Cost of treatment and services, remission rate, health outcome
144	Le et al., 2017 [157]	Australia	N/A		Outpatient (adolescents, both sexes)	To evaluate the cost-effectiveness of FBT compared to adolescent-focused individual therapy or no intervention	Modelling (statistical)	Incremental cost effectiveness
145	Le Grange et al., 2012 [158]	USA	121		Outpatient (adolescents, both sexes)	To identify treatment moderators and mediators of remission for adolescents with AN via FBT and individual adolescent focused therapy	RCT	Severity of ED pathology using the EDE V12.0; depressive symptoms using the BECK Depression Inventory (BDI) and body weight
146	Le Grange et al., 2015 [159]	USA	130		Outpatient (adolescents, both sexes)	To compare the relative efficacy of therapies of FBT for adolescent bulimia nervosa and CBT adapted for adolescents	RCT	Abstinence of Binge-eating rates and purging for 4 weeks before assessment using the EDE
147	Le Grange et al., 2016 [160]	Australia	107		Outpatient (adolescents, both sexes)	To compare the relative efficacy of FBT and parent-focused treatment	RCT	Remission defined as ≥ 95% of median body mass index and Eating Disorder Examination Global Score within 1SD of community norms
148	Levinson et al., 2015 [161]	USA	36		Outpatient/inpatient (mixed cohort, both sexes)	To examine D-cycloserine-facilitation of exposure therapy would increase body mass index in patients with AN	RCT	Body Mass Index (BMI), anxiety using the Subjective Units of Distress Scale (SUDS)
149	Linardon et al., 2017 [162]	Worldwide		27	Outpatient (adult, both sexes)	To examine the empirical status of third-wave behaviour therapies	Systematic review/meta-analysis (combined)	ED Psychopathology, EDE global score, remission/recovery, binge eating, shape concern, depression, self-esteem
150	Linardon et al., 2017 [163]	Worldwide		79	Community (mixed cohort, both sexes)	To examine the efficacy of CBT on eating disorders	Systematic review/meta-analysis (combined)	The efficacy of CBT for ED
151	Linardon et al., 2017 [164]	Worldwide		26	Mixed (mixed cohort, both sexes)	To examine the efficacy of psychotherapy for BN on depressive symptoms in the short- and long-term	Systematic review/meta-analysis (combined)	Efficacy of psychotherapy on symptoms of depression
152	Linardon et al., 2020 [165]	Australia		36	Inpatient and outpatient (mixed cohort, both sexes)	To conduct an updated meta-analysis on the efficacy of e-mental health interventions for treating and preventing eating disorders	Systematic review/meta-analysis (combined)	Efficacy of e-mental health interventions paying attention to trial quality and outcomes
153	Linardon et al., 2022 [166]	Australia	332		Outpatient (adults, both sexes)	To develop and evaluate a smartphone app based on the principles and techniques of transdiagnostic CBT for eating disorders	RCT	Global levels of ED psychopathology; other ED symptoms, impairment and distress
154	Linardon, 2018 [167]	Australia		29	Community (adult, both sexes)	To examine whether CBT can modify dietary restraint and attitudes towards shape and weight	Meta-analysis	Dietary restraint and dysfunctional attitudes towards shape and weight
155	Linardon, Kothe and Fuller-Tyszkiewicz, 2019 [168]	Worldwide		34	Outpatient (adult, both sexes)	To examine the effects of psychotherapy for bulimia nervosa and binge-eating disorder on self-esteem	Meta-analysis	The effects of psychotherapy for BN and BED on self‐esteem improvement
156	Linardon, Messer and Fuller-Tyszkiewicz, 2018 [167]	Australia		27	Outpatient (adult, both sexes)	To conduct a meta-analysis of nonrandomized studies of cognitive behaviour therapy (CBT) for EDs	Meta-analysis	Abstinence rates of binge-eating
157	Linnet et al., 2022 [169]	Denmark	143		Community (adults, both sexes)	To investigate the association between the number of words per message and adherence in a text based iCBT program for BED	Quasi-experimental (intervention)	BED symptoms, depressive symptoms
158	Lock et al., 2010 [170]	USA	121		Outpatient (adolescents, both sexes)	To evaluate the relative efficacy of FBT and adolescent-focused individual therapy for adolescents with AN	RCT	Full remission or partial remission rates; mean global Eating Disorder Examination Score within one SD of published means
159	Lock et al., 2015 [171]	USA	45		Outpatient (adolescents, both sexes)	To examine the feasibility and effects of treatment aimed at enhancing parental self-efficacy related to re-feeding skills in poor early responders to FBT	RCT	Recruitment and attrition rates, percentile body weight, Therapy Suitability and Patient Expectancy (TSPE) Scale, Psychopathology (EDE)
160	Loeb et al., 2012 [172]	N/A		N/A	Outpatient (adolescents, both sexes)	To describe the transdiagnostic theory and application of FBT for children and adolescents with EDs	Review (narrative)	Not defined
161	Low et al., 2021 [173]	Worldwide		6	Inpatient and outpatient (mixed cohort, both sexes)	To examine the efficacy of virtual reality-enhanced CBT in the treatment of binge-purging type EDs compared to CBT	Meta-analysis	BMI and frequency of binges and/or purges; body satisfaction; situation induced body dissatisfaction
162	Lydecker and Grilo, 2016 [174]	USA	344		Outpatient (mixed cohort, both sexes)	To examine differences in child ED behaviours and parental feeding practices between parents exhibiting AN, BN, binge-eating/purging disorder with parents reporting no ED characteristics	Cross-sectional	BMI, Eating Disorder Examination (EDE), Child Feeding Questionnaire (CFQ)
163	Lynch et al., 2010 [175]	USA	123		Outpatient (mixed cohort, both sexes)	To conduct an incremental cost-effectiveness analysis of a CBT guided self-help intervention to treat recurrent BE compared to TAU	RCT	Binge-free days and quality-adjusted life years (QALYs); cost to patients and the health plan
164	Macdonald et al., 2012 [176]	Worldwide		13	Outpatient (adults, both sexes)	To examine the context and effectiveness of motivational interviewing and Motivational Enhancement Therapy in patients or carers of people with ED's to identify limitations, difficulties and research needs	Review (systematic)	Varied
165	MacDonald, Trottier and Olmsted, 2017 [177]	Canada	104		Outpatient (adults, both sexes)	To examine whether rapid change in emotion regulation predicted treatment outcomes, beyond the effects of RSBC	Repeated measure (without follow-up)	Eating Disorder Examination Questionnaire (EDE-Q), Self-monitored ED behaviours, Difficulties in Emotion Regulation Scale (DERS), Beck Depression Inventory (BDI)
166	Madden et al., 2015 [178]	Australia	69		Outpatient (adolescents, both sexes)	To Identify whether early weight gain in family-based treatment (FBT) predicted greater weight and remission at end of FBT and 12-month follow-up	RCT	Weight gain
167	Magson, Handford and Norberg, 2021 [179]	Worldwide		20	Outpatient (mixed cohort, both sexes)	To determine the need for higher quality research that utilizes larger samples/uniform outcome measures that are more strongly grounded in theory	Review (systematic)	Changes in eating behaviours, cue reactivity and/or CS-US expectancies from pre- to post-treatment
168	Mallorqui-Bague et al., 2018 [180]	Spain	507		Outpatient (adult, females)	To explore Emotion Regulation difficulties and to assess pre-treatment and post-treatment changes among ED types	Longitudinal (< 5 years)	Pre-treatment and post-treatment changes among ED types
169	Månsson, Parling and Swenne, 2016 [181]	Sweden	47		Outpatient (adolescents, both sexes)	To study the effect of defined parental interventions on restrictive EDs in adolescents	Repeated measures (without follow up)	Weight change and Eating Disorder Examination Questionnaire at 1 week, 1 month, 3 months; EDE-Q score at start of treatment and at 3 months
170	Marco, Perpina and Botella, 2013 [182]	Spain	34		Outpatient (mixed cohort, females)	To compare CBT for EDs with/without a component for body image treatment using Virtual Reality techniques	RCT	Body-image; Body Attitude Test (BAT), Body Image Automatic Thoughts Questionnaire (BIATQ), Body Areas Satisfaction Scale (BASS), Situational Inventory of Body-Image Dysphoria (SIBID), The Bulimic Investigatory Test, Edinburgh (BITE), The Eating Attitudes Test (EAT)
171	Marcos and Cantero, 2009 [183]	Spain	98		Outpatient (mixed cohort, females)	To assess social support dimensions in patients with ED, looking at diagnosis, socio-demographic and clinical characteristics, and self-concept	Cross-sectional	Social support assessment
172	Masheb, Grilo and Rolls, 2011 [184]	USA	50		Outpatient (adult, both sexes)	To examine a dietary approach for producing weight loss in obese patients with BED who also received CBT	RCT	Percentage of participants achieving at least a 5% weight loss using BMI; Binge-remission, energy density, psychopathology using EDE
173	Mason et al., 2017 [185]	USA	171		Community and outpatient (adult, both sexes)	To examine baseline and longitudinal associations between eating-related and psychosocial variables and dimensions of weight QOL	RCT	Body mass index (BMI), ED psychopathology, and psychosocial factors
174	Matthews et al., 2018 [186]	USA	51		Community (mixed cohort, both sexes)	To examine the relation between caregiver illness perceptions about AN, symptom severity indicators, and caregiver burden in a sample of medically hospitalized youth with AN	Cross-sectional	Brief Illness Perceptions Questionnaire (Brief IPQ), Eating Disorder Symptom Impact Scale (EDSIS), Eating Disorder Examination-Questionnaire (EDE-Q), Children's Depression Inventory-2 Short Form (CDI-2-S), Multidimensional Anxiety Scale for Children-Second Edition (MASC-2)
175	McClay et al., 2016 [187]	UK	253		Mixed (mixed cohort, both sexes)	To investigate attitudes towards online self-help for ED's and the support required	Cross-sectional	Attitudes towards on-line self-help
176	McIntosh et al., 2011 [188]	New Zealand	135		Outpatient (adult, females)	To identify any evidence of a conditioned inoculation from exposure treatment compared with relaxation training in long-term abstinence from binge eating	RCT	Long-term abstinence rates and frequency of purging from binge-eating over 5 years
177	McIntosh et al., 2016 [189]	New Zealand	112		Outpatient (adult, females)	To compare CBT with CBT; schema therapy, and appetite-focused CBT	RCT	Frequency of binge-eating; some behavioural and psychological aspects
178	Mercado et al., 2021 [190]	UK		12	Mixed (adult, both sexes)	To examine randomised controlled trials (RCTs) comparing the efficacy of MBIs with control groups primarily encouraging either dietary or exercise-based behavioural change in individuals with overweight/obesity and/or binge eating disorder (BED)	Systematic review/meta-analysis (combined)	Changes in body mass (BMI or weight), mindfulness scores, BED symptoms
179	Moberg et al., 2021 [191]	Norway		62	Mixed (adults, both sexes)	To shed light on how these two specific and conceptually different treatment approaches work for patient samples with varying presentations of EDs in both RCTs and observational studies	Review (meta-analysis)	ED psychopathology, ED remission
180	Moghmi, Davis and Rotondi, 2021 [192]	Worldwide		3	Community (adults, both sexes)	To determine the effectiveness of eHealth treatments in adults diagnosed with full or subthreshold BED	Systematic review/meta-analysis (combined)	Objective binge episodes, BMI, EDE-Q, shape concern, weight concern
181	Monteleone et al., 2022 [193]	Worldwide		59	Mixed (mixed cohorts, both sexes)	To conduct a review accounting for effect modifiers and confounding factors, and conducting analyses by age group, type of intervention, setting, control condition, ED diagnosis (i.e. AN, BN, BED, EDNOS), and mode of treatment delivery	Meta-analysis	ED-specific behaviours, neuropsychological functioning, eating disorder-specific psychopathology, functioning and quality of life, general psychiatric symptoms, global course of the disease, weight or BMI
182	Munsch, Meyer and Biedert, 2012 [194]	Switzerland	48		Community (adult, both sexes)	To assess the long-term efficacy of Cognitive-Behavioural Treatment (CBT) and Behavioural Weight-Loss-Treatment (BWLT) in patients with BED and to identify potential predictors of long-term treatment success	RCT	ED psychopathology, BMI, negative affect, therapeutic process variables and contentment with therapy
183	Murphy et al., 2012 [195]	N/A		N/A	Outpatient (mixed cohort, both sexes)	To consider, review and discuss the rationale for using Interpersonal psychotherapy to treat patients with EDs	Review (narrative)	Evidence supporting the use of IPT
184	Murray et al., 2015 [196]	USA	35		Outpatient/inpatient (adolescents, both sexes)	To investigate the efficacy of integrating FBT and dialectical behaviour therapy in treating adolescent bulimia nervosa	Repeated measures (no follow up)	Core bulimia nervosa pathology; emotional regulation difficulties; parental measures of self-efficacy
185	Onnink et al., 2022 [197]	USA	22	674	Mixed (mixed cohorts, both sexes)	To review the evidence of Acceptance and Commitment Therapy (ACT) for EDs through January of 2022	Review (systematic)	ED behaviour, body image, weight
186	O’Reilly et al., 2014 [198]	Worldwide		21	Outpatient (adults, both sexes)	To conduct a literature review to determine the effectiveness of mindfulness-based interventions for treating binge eating, emotional eating and external eating	Review (systematic)	Changing obesity-related eating behaviours, specifically binge eating, emotional eating and external eating
187	Pacanowski et al., 2018 [199]	USA	189		Outpatient (adult, both sexes)	To characterise factors that promote or inhibit weight loss in individuals with BED and obesity	RCT	BMI, Eating Disorder Examination (EDE), Three Factor Eating Questionnaires (TFEQ)
188	Palavras et al., 2017 [200]	Worldwide		19	Outpatient (adult, both sexes)	To review efficacy of psychological therapies for BN and BED in reducing binge frequency and weight	Meta-analysis	Binge-eating frequency or binge remissions; weight or BMI at end of treatment and at 1 year follow up; treatment completion rates
189	Palavras et al., 2021 [201]	Brazil	98		Outpatient (adults, both sexes)	To investigate the efficacy and safety of introducing a weight loss intervention to the treatment of people with disorders of recurrent binge eating and a high BMI	RCT	Sustained weight loss at 12 months, ED psychopathology
190	Parling et al., 2016 [202]	USA	43		Outpatient (adult, both sexes)	To compare 19 sessions of ACT with TAU, after 9 to 12 weeks of day care, regarding recovery and risk of relapse up to 5 years	RCT	Body Mass Index (BMI) and specific eating psychopathology
191	Paslakis et al., 2017 [203]	Germany	30		Outpatient (females)	To determine if the novel virtual reality paradigm may serve as a therapeutic tool for exposure and habituation of the urge of acutely engaging in physical activity in patients with EDs	Cross-sectional	Cognitive, emotional, and behavioural aspects of the acute urge to move, leptin levels
192	Peat et al., 2017 [204]	Worldwide		30	Community (adult, both sexes)	To expand the literature for BED studies to include a review of comparative effectiveness	Review (systematic)	Abstinence, frequency, weight
193	Pennesi and Wade, 2016 [3]	Worldwide		54	Community (adult, females)	To identify existing models for disordered eating and to identify those models which have helped inform the development of interventions for disordered eating	Review (systematic)	Disordered eating models (existing and those that informed the development of interventions for disordered eating)
194	Pepin and King, 2016 [205]	Australia	77		Community (mixed cohort, both sexes)	To examine if Collaborative Care Skills Training workshops by facilitators trained in its delivery could lead to improve the well-being, coping strategies and problem-solving skills of carers of someone with an ED	Repeated measures (with follow up)	Coping strategies, expressed emotion (EE), burden, distress, confidence in capacity to change, accommodating and enabling of ED behaviour
195	Pepin and King, 2013 [206]	Australia	15		Community (mixed cohort, both sexes)	To present the results of a RCT measuring the efficacy of a video-based skills training to decrease burden and psychological distress in caregivers of inpatients treated for an ED in specialized hospital units	Repeated measures (with follow up)	General psychological health (psychological distress), adaptive and maladaptive coping, expressed emotion (critical comments and emotional over involvement), burden experienced by carers and perceptions of impact of caring for someone with an ED (nutritional difficulties, guilt, manifestation of dysregulated behaviour, social isolation)
196	Perpina et al., 2013 [207]	Spain	59		Outpatient (adult, females)	To examine the clinical validation of a Virtual Reality Environment designed to normalize eating patterns in EDs	Control trial	The Reality Judgment and Presence Questionnaire (RJPQ); The revised version of the ITC-Sense of Presence Inventory (ITC-SOPI); Beck Depression Inventory (BDI-II); Beck Anxiety Inventory (BAI); The Restraint Scale (RS)
197	Peterson et al., 2017 [208]	USA	80		Outpatient (adult, both sexes)	To examine the indirect effects of Integrative Cognitive-Affective Therapy and CBT-E on BN treatment outcome through the variables of emotion regulation, self-directed behaviour, and self-discrepancy	RCT	EDE Binge-eating and Purging frequency as well as global score at end of treatment and 4-month follow up
198	Peterson et al., 2020 [209]	USA	112		Outpatient (adult, both sexes)	To compare Integrative Cognitive-Affective Therapy with an established cognitive-behavioural guided self-help treatment using standard and ecological momentary assessment outcome measures	RCT	Frequency of binge eating as measure by the EDE and EMA (hypothesised maintenance mechanisms also assessed)
199	Philipp et al., 2021 [210]	Austria	98		Outpatient (adolescent, both sexes)	To evaluate the effectiveness of a caregivers’ skills training program on adolescents with AN delivered as workshops or online	Repeated measures (without follow up)	BMI, eating psychopathology, motivation to change, emotional and behavioural problems and quality of life
200	Pinto-Gouveia et al., 2017 [211]	Portugal	59		Outpatient (adult, females)	To test the acceptability and efficacy of a psychological program based on psychoeducation, mindfulness, and self-compassion for obese or overweight women with BED	Longitudinal (< 5 years)	EDE 16.0, Binge Eating Scale, Beck Depression Inventory, Shamer Scale, Obesity-Related Well-Being Questionnaire, Body Image-Acceptance and Action Questionnaire
201	Pisetsky et al., 2015 [212]	USA	190		N/A	To examine whether perceptions of group dynamics early in treatment predicted ED outcomes in adults with BED in 15 CBT group sessions with differing levels of therapist involvement (led, assisted, and self-help)	RCT	EDE Global Score, Number of OBE in past 21 days
202	Pittock, Hodges and Lawrie, 2018	Worldwide		5	Outpatient (adult, females)	To review if internet-delivered CBT as a possible treatment for patients with bulimic symptoms	Review (systematic)	Binge-eating and purging
203	Polnay et al., 2014 [213]	Worldwide		10	Mixed (mixed cohort, both sexes)	To evaluate how group therapy for BN compares with individual therapy, no treatment, or other therapies, in terms of remission from binges and binge frequency	Systematic review/meta-analysis (combined)	Remission from binges and binge-frequency
204	Poulsen et al., 2014 [214]	Denmark	70		Outpatient (adult, both sexes)	To compare psychoanalytic psychotherapy and CBT in the treatment of BN	RCT	Eating disorder examination interview at baseline, after 5 months and after 2 years
205	Puls, Schmidt and Hilbert, 2019 [215]	Germany	64		Outpatient (young people, both sexes)	To examine therapist adherence and therapeutic alliance and their associations in CBT for adolescents with BED	RCT	Variability of adherence and alliance) explained by treatment module, patient, and therapist)
206	Quadflieg et al., 2017 [216]	Germany	285		Community (mixed cohort, both sexes)	To investigate the feasibility and usefulness of an online information and skills development intervention for carers of individuals with AN and individuals with AN	RCT	Eating Disorder Symptom Impact Scale, Accommodation and Enabling Scale and General Health Questionnaire-12 at baseline and 3-month follow up
207	Rahmani et al., 2018 [217]	Iran	60		Outpatient (adult, females)	To determine the effect of dialectical behaviour therapy on BED, difficulties in emotion regulation, and BMI of patients suffering from BED and overweight	RCT	BMI, binge eating and emotion regulation ability
208	Ramklint et al., 2012 [218]	Sweden	89		Mixed (mixed cohort, both sexes)	To describe the implementation and effectiveness of the first step, guided self-help of a stepped-care model of CBT for patients with bulimic symptoms	Case series	Decrease in bulimic symptoms
209	Raykos et al., 2014 [219]	Australia	112		Outpatient (adult, both sexes)	To assess therapeutic alliance over the course of CBT-E for patients with a diagnosis of BN or atypical BN	Cross-sectional	Alliance, treatment retention and outcome
210	Raykos et al., 2014 [220]	Australia	406		Mixed (mixed cohort, both sexes)	To examine whether baseline interpersonal problems differed across ED diagnoses and subtypes	Cross-sectional	Eating-disorder psychopathology and socialising ability
211	Ricca et al., 2010 [221]	Italy	144		Outpatient (adult, both sexes)	To compare individual and group CBT for BED	RCT	Recovery at 3-year follow-up, weight-loss, treatment resistance, relapse, and diagnostic change
212	Richards et al., 2018 [222]	Worldwide		30	Outpatient (adolescents, both sexes)	To review the literature reporting outcomes of augmentative FBT interventions for adolescents with restrictive EDs	Review (systematic)	Body weight and/or ED symptoms at end of treatment
213	Rieger et al., 2010 [223]	N/A		N/A	N/A	To develop a theoretical model of interpersonal psychotherapy in the context of EDs	Review (narrative)	Social Evaluation
214	Riesco et al., 2018 [224]	Spain	176		Outpatient (adult, both sexes)	To examine features of Other Specified Feeding or EDs subtypes, CBT responses and clinical predictors of therapy outcome	Case series	Eating related psychopathological and personality measures
215	Robinson et al., 2013 [225]	Canada	49		Outpatient (adolescents, both sexes)	To examine parent and adolescent outcomes of FBT and the role of parental self-efficacy in adolescent ED, depressed mood and anxiety symptoms	Repeated measures (with follow up)	Changes in ED symptomatology, as well as in ratings of depressed mood and anxiety symptoms
216	Romero-Martinez, Ruiz-Robledillo and Moya-Albiol, 2016 [226]	Spain	54		Community (adult, both sexes)	To characterize whether caregivers of individuals with EDs show declarative memory impairments compared to non-caregiver’s caregivers	Cross-sectional	Memory impairment Rey Auditory Verbal Learning Test (RAVLT), General Health Questionnaire (GHQ-28), Level of testosterone (T)
217	Rossi et al., 2021 [227]	Italy	141		Outpatient (adult, females)	To evaluate the role of its variation as a possible mediator of the efficacy of enhanced CBT on classic ED symptomatology, including body uneasiness	Longitudinal (< 5 years)	BMI and psychometric scales (EDE-Q6, BUT-A, IDEA, SCL 90-R, GSI)
218	Rozakou-Soumalia, Dȃrvariu and Sjögren, 2021 [228]	Denmark		11	Mixed (adult, both sexes)	To study the effect of DBT in ED and specifically the effect of DBT on ER in comparison to a control group and to investigate the effect on general psychopathology and Body Mass Index (BMI)	Systematic review/meta-analysis (combined)	Emotion regulation, ED psychopathology, BMI
219	Sanchez-Ortiz et al., 2011 [229]	UK	76		Outpatient (adult, females)	To evaluate if Internet-based CBT may be able to bridge the gap of female students who do not access effective treatment	RCT	ED outcomes were assessed with the ED examination (EDE); depression, anxiety and quality of life
220	Scanferla et al., 2022 [230]	France	169		Outpatient (adult, both sexes)	To test this novel intervention assuming it would be an innovative “first fast” step into care, facilitating access and commitment in specialised treatment of users with newly diagnosed eating disorders	Quasi-experimental (intervention)	ED psychopathology, subjective satisfaction
221	Schag et al., 2019 [231]	Germany	80		Mixed (mixed cohort, both sexes)	To compare the results of a cognitive behavioural group intervention focusing on impulsive eating with a randomised control group	RCT	Binge-eating episodes; eating pathology, depression, general impulsivity and body-mass-index (BMI)
222	Schlegl et al., 2015 [232]	Worldwide		45	Outpatient (mixed cohort, both sexes)	To evaluate the potential of technology-based interventions in EDs (AN and BN) for prevention and treatment, and also for carers of ED patients	Review (systematic)	Varied
223	Schlup, Meyer and Munsch, 2010 [233]	Switzerland	76		Outpatient (adult, females)	To compare treatment outcomes of short-term and long-term CBT for BED in a non-randomized comparison and to identify moderators of treatment outcome	Repeated measures (with follow up)	Remissions from binge-eating based on EDE, number of OBEs (EDE), associated disorder psychopathology (4EDE sub scales) and BMI
224	Schwarte et al., 2017 [234]	Germany	296		Community (Mixed cohort, both sexes)	To assess the levels of expressed emotions and depressive symptoms found in caregivers of patients with AN	Longitudinal (< 5 years)	Levels of Expressed Emotions (EE) and Depressive Symptoms (DS)
225	Shimshoni et al., 2020 [235]	USA	14		Community (adult, both sexes)	To assess the feasibility, acceptability, treatment-satisfaction, and preliminary efficacy of Supportive Parenting for Anxious Childhood Emotions adapted for avoidant/restrictive food intake disorder	Repeated measure (without follow-up)	The feasibility, acceptability, treatment-satisfaction, and preliminary efficacy of Supportive Parenting for Anxious Childhood Emotions
226	Slade et al., 2018 [236]	Worldwide		21	Community (adult, both sexes)	To compare the effectiveness of pharmacological, psychological and a combination of treatments to identify the most effective for BN	Meta-analysis	Full remission at end of treatment
227	Sodersten et al., 2017 [237]	Worldwide		N/A	Mixed (adult, both sexes)	To examine the science and evidence supporting CBT for the treatment of BN and other EDs	Review (narrative)	Remission rate, relapse rate
228	Solmi et al., 2021 [238]	Worldwide		16	Outpatient (adult, both sexes)	To compare stand-alone psychological interventions for adult outpatients with AN with a specific focus on BMI, ED symptoms, and all-cause dropout rate	Systematic review/meta-analysis (combined)	BMI, Frequency of Eating Disorder Symptoms, Clinical symptoms and all-cause drop-out rate
229	Spielmans et al., 2013 [239]	Worldwide		53	Community (adult, both sexes)	To conduct meta-analysis comparing psychological treatments for BN and BED and the role of moderating variables	Meta-analysis	Therapist allegiance, supervision, pretraining, homework
230	Steele, Bergin and Wade, 2011 [240]	Australia	87		Mixed (mixed cohort, both sexes)	To examine predictors of guided self-help treatment outcome in BN	RCT	Stress, Eating Disorder-Related Automatic Thoughts, Frequency of Binge episodes
231	Stefanini et al., 2019 [241]	Italy	97		Community (adult, both sexes)	To study the factors affecting caregivers living with someone with an ED	Cross-sectional	The accommodation and enabling scale for eating disorders (AESED), The family questionnaire (FQ), The depression, anxiety and stress scale (DASS-21)
232	Stefini et al., 2017 [242]	Germany	81		Outpatient (adolescent, females)	To compare CBT and psychodynamic therapy for the treatment of BN in female adolescents	RCT	Rate of remission defined as a lack of the Diagnostic and Statistical Manual of Mental Disorders (DSM-IV) diagnosis at the end of therapy
233	Stein et al., 2013 [243]	USA	69		Outpatient (adult, females)	To determine if findings of a randomized trial of an identity intervention programme designed to build new positive self-schemas that are separate from other conceptions of the self in memory as the means to promote improved health in women diagnosed with EDs are reported	RCT	ED symptoms at 1-month, 6-months, 12-months post intervention
234	Steinberg et al., 2023 [244]	USA	210		Community (young people, both sexes)	To describe our FBT + treatment approach, highlight preliminary clinical outcomes, and discuss implications for the future of eating disorder treatment	Cross-sectional	Weight, ED symptoms, depression and anxiety symptoms, caregiver burden and self-efficacy, treatment acceptability and satisfaction
235	Steinglass et al., 2011 [245]	USA		N/A	Outpatient (mixed cohort, both sexes)	To review data on anxiety in AN, the relationship between anxiety disorders and AN, and the use of Exposure and Response Prevention in treatment	Review	Varied
236	Steinglass et al., 2014 [246]	USA	32		Inpatient (adult, both sexes)	To evaluate AN Exposure and Response Prevention (AN-EXRP) as an adjunctive strategy to improve eating behaviour during weight restoration	RCT	Average test meal caloric intake
237	Stice et al., 2019 [247]	USA	84		Outpatient (adult, females)	To evaluate a dissonance-based group ED treatment	RCT	ED diagnosis; ED symptom change; level of dissonance about affirming the thin-ideal
238	Stile-Shields et al., 2013 [248]	USA	64		Outpatient (adult, females)	To investigate the strength and role of therapeutic alliance by comparing CBT and Specialist Supportive Clinical Management for the treatment of AN	RCT	Perception of the quality of the therapeutic relationship, eating disorder (ED) symptomatology, and depressive symptomatology
239	Stiles-Shields et al., 2012 [249]	N/A		N/A	Outpatient (adolescent, both sexes)	To provide a critical literature overview focusing on FBT especially adolescents	Review (other)	Not defined
240	Strandskov et al., 2017 [250]	Sweden	92		Community (adult, both sexes)	To investigate the outcome of tailored and ACT-influenced, Internet CBT treatment for ED psychopathology, and the relation between knowledge acquisition and outcome	RCT	Eating disorder symptoms and body shape dissatisfaction
241	Striegel-Moore et al., 2010 [251]	USA	123		Outpatient (mixed cohort, both sexes)	To evaluate whether a manual-based guided self-help form of CBT, in 8 sessions over a 12-week period, is more effective than TAU	RCT	Patient Health Questionnaire eating disorder module (PHQ-ED); Eating Disorder Examination (EDE); Structured Clinical Interview for DSM-IV; Beck Depression Inventory
242	Swenne, Parling and Ros, 2017 [252]	Sweden	201		Outpatient/inpatient (adolescent, both sexes)	To investigate the 1-year outcome of a family-based intervention programme with defined and decisive interventions at the start of treatment	Repeated measures (with follow up)	BMI and psychometric measures (EDE-Q)
243	Tatham, 2011 [253]	N/A		N/A	Outpatient (adult, both sexes)	To consider theoretical and empirical findings in terms of the clinical applicability of imagery-based techniques and their ability to enhance CBT	Review (narrative)	Varied
244	Tecuta and Tomba, 2018 [254]	Italy	60		Outpatient (adult, females)	To investigate subjective incompetence and its association with psychological distress and well-being	Repeated measure (without follow-up)	Subjective incompetence
245	ter Huurne et al., 2015 [255]	Netherlands	214		Community (adult, females)	To evaluate the effects of a Web-based CBT using intensive asynchronous therapeutic to improve ED psychopathology, reduce body dissatisfaction and related health problems among ED patients	RCT	ED psychopathology; body dissatisfaction; physical health, mental health, self-esteem, quality of life, social functioning
246	ter Huurne et al., 2021 [256]	Netherlands	212		Community (adult, females)	To report on a 1-year follow-up study into a web-based CBT for female patients with ED	RCT with 1-year follow-up	ED psychopathology, body dissatisfaction, BMI, physical health, mental health, self-esteem, quality of life, social functioning
247	Thomas et al., 2017 [257]	N/A		N/A	Outpatient/inpatient (mixed cohort, both sexes)	To summarise what is known about avoidant/restrictive food intake disorder and to introduce a three-dimensional model	Review (other)	Not defined
248	Thomas et al., 2021 [258]	USA	17		Outpatient (adolescent, both sexes)	To evaluate feasibility, acceptability, and proof-of-concept for CBT for avoidant/restrictive food intake disorder in children and adolescents	Repeated measures (without follow-up)	Feasibility, acceptability, and proof-of-concept for CBT
249	Thomas, Wons and Eddy, 2018 [259]	N/A		N/A	Outpatient/inpatient (mixed cohort, both sexes)	To review the literature on avoidant/restrictive food intake disorder treatment and highlight a novel cognitive-behavioural approach	Review (other)	Not defined
250	Thompson-Breener, Boisseau and Satir, 2010 [260]	USA	120		Outpatient (adolescent, both sexes)	To undertake a naturalistic study of the treatment and outcome of adolescents with EDs in the community	Case series	Observation
251	Thompson-Brenner et al., 2015 [261]	USA	43		Community (adult, females)	To compare changes in purging, depression, and cognitive ED symptoms for associations with BN remission	Quasi-experimental (intervention)	Percentage change in purging frequency and percentage change in BDI score and Rate of Remission
252	Thompson-Brenner et al., 2016 [262]	USA	50		Outpatient (adult, both sexes)	To investigate the relative effects, predictors, and moderators of CBT for BN with personality and mood/anxiety disorders	RCT	Frequency of remission from binge-eating and purging at termination; severity of affective/interpersonal problems using EDE scores
253	Thompson-Brenner et al., 2021 [263]	USA	3108		Community (mixed cohorts, both sexes)	To examine the effect of the Renfrew Unified Treatment for Eating Disorders and Comorbidity (UT) implementation across 5 years of treatment delivery	Longitudinal (5-years)	ED symptom severity, depressive symptoms, experiential avoidance, anxiety sensitivity, mindfulness
254	Traviss, Heywood-Everett and Hill, 2011 [264]	UK	81		Outpatient (mixed cohort, both sexes)	To evaluate a CBT-based pack delivered by trained mental health professionals in 6 sessions over 3 months	RCT	Eating disorder psychopathology (EDE-Q), key behavioural features and global distress (CORE)
255	Traviss-Turner, West and Hill, 2017 [265]	Worldwide		30	Outpatient (mixed cohort, both sexes)	To establish the effectiveness of guided self-help for reducing global ED psychopathology and abstinence from BE, compared with controls	Systematic review/meta-analysis (combined)	Level of psychopathology and abstinence from binge eating
256	Treasure et al., 2015 [8]	Worldwide		N/A	N/A	To map the possibility of new treatment approaches for EDs	Review (narrative)	Primary and secondary symptoms seen in the enduring stage of ED
257	Turner, Bryant-Waught and Marshall, 2015 [266]	UK	94		Outpatient (adult, both sexes)	To explore the impact of early symptom change and the early therapeutic alliance on treatment outcome in CBT for EDs	Longitudinal (< 5 years)	Early symptom change (cognitive and behavioural) and the early therapeutic alliance on treatment outcome
258	Valenzuela et al., 2018 [267]	USA	110		Outpatient (adolescent, both sexes)	To examine the effect of FBT for BN and CBT on depressive symptoms and self-esteem in adolescents with BN	RCT	Depressive symptoms and self-asteem; Beck Depressive Inventory (BDI; Panel A) and Rosenberg Self‐Esteem Scale (RSES; Panel B)
259	Vancampfort et al., 2014 [268]	Belgium	34		Outpatient (adult, both sexes)	To explore the associations between changes in number of binges, physical activity, physical fitness, physical self-perception and QOL after 6-month physical activity counselling and CBT program in patients with BED	Repeated measures (without follow up)	Frequency of binges was assessed using the Eating Disorder Examination (EDE); Physical fitness: the 6-min walk test (6MWT); Health related quality of life: the MOS 36-item Short Form Health Survey (SF-36); Physical activity: Baecke Physical Activity Questionnaire; The Physical Self Perception Profile (PSPP);
260	Vancampfort et al., 2014 [269]	Belgium	100		Outpatient (adult, both sexes)	To compare the mental and physical health related QOL of 40 obese persons with BED with 20 age, gender and BMI matched obese persons without BED and 40 age and gender matched non-obese volunteers	Cross-sectional	Mental and physical health-related quality of life (HRQL)
261	Vaz, Conceicao and Machado, 2013 [270]	Portugal	42		Mixed (mixed cohort, both sexes)	To test the effectiveness of a cognitive-behavioural guided self-help treatment program for BN and similar disorders	Repeated measures (with follow up)	Eating Disorder Examination questionnaire (EDE-Q) score; Outcome-Questionnaire (OQ-45) score and Back Depression Inventory (BDI) score
262	Vaz, Conceição and Machado, 2014 [271]	Portugal	42		Outpatient (adult, females)	To investigate the sessions/time required for a clinical change with a guided self-help CBT treatment, to assess the predictive value of early response and other potential predictors of end-of-treatment clinical status	Case study	Structured Clinical Interview for DSM-IV (SCID), Eating Disorders Examination (EDE), Eating Disorders Examination Questionnaire (EDE-Q), Outcome Questionnaire (OQ), Beck Depression Inventory (BDI)
263	Vella-Zarb et al., 2014 [272]	Canada	45		Mixed (mixed cohort, both sexes)	To build on current research by comparing Motivational Interviewing with psychoeducation, each as a prelude to self-help treatment for BE	RCT	Readiness to change and confidence in ability to control binge eating
264	Vocks et al., 2010 [273]	Worldwide		38	Mixed (mixed cohort, both sexes)	To compute and compare mean effects of various treatments for BED	Meta-analysis	Varied
265	Vogel, Singh and Accurso, 2021 [274]	USA		50	Community (adolescent, both sexes)	To summarize and critically analyse the current literature on the feasibility, acceptability, effectiveness, and efficacy of CBT and DBT for adolescent eating disorders, and then proposes areas of future research	Systematic review/meta-analysis (combined)	AN outcomes, BN outcomes, BED outcomes, transdiagnostic outcomes
266	von Brachel et al., 2014 [275]	Germany	179		Community (adult, females)	To identify predictors of dropout from a Web-based 6-session program to enhance motivation to change for women with AN, BN, or related subthreshold eating pathology	Quasi-experimental (intervention)	ED pathology measured by EDE-Q; Motivation to change using URICA; Depressive Mood using HSCL-25
267	Wade et al., 2021a [276]	Australia	120		Outpatient (adult, both sexes)	To identify latent classes of trajectory of change in BMI between the initial and thirteenth session of outpatient treatment for adult AN and identify the association with outcome	Repeated measures (without follow up)	Body weight, ED psychopathology (EDE)
268	Wade et al., 2021b [277]	Australia	98		Outpatient (young/adult, both sexes)	To compare the efficacy of two forms of CBT-ED for patients with a BMI of more than 17.5—CBT-T and an expanded (10-session) CBTm—and to conduct an exploratory investigation of moderators	RCT	Global eating psychopathology, clinical impairment, depression, anxiety and stress, remission and good outcome, body avoidance, body checking, motivation
269	Wagner et al., 2013 [278]	Austria	155		Mixed (mixed cohort, both sexes)	To evaluate in a RCT the long-term effectiveness of internet guided self-help compared with conventional guided bibliotherapy in females with BN	RCT	Frequency of binge eating, vomiting and fasting
270	Wagner et al., 2015 [279]	Austria	126		Mixed (mixed cohort, both sexes)	To determine predictors of good long-term outcome and drop-out, in order to identify patients for whom these interventions are most suitable	RCT	Depressive symptomatology using BDI; general psychopathology using SIAB-EX; eating disorder inventory (EDI-2); temperament and character using TSI-R
271	Wagner et al., 2016 [280]	Germany	139		Outpatient (adult, both sexes)	To examine the efficacy of an Internet-based cognitive-behavioral intervention and to examine the stability of treatment effects over 12 months	RCT	Binge eating episodes, Eating Disorder Examination–Questionnaire (EDE-Q), Beck Depression Inventory (BDI), Symptom Checklist-90-Revised (SCL-90-R), body weight and BMI
272	Walker and Bryant, 2013 [281]	N/A		27	N/A	To synthesize findings from studies of the belief that people who have endured and overcome a psychiatric disability can offer useful support, encouragement, and hope to their peers	Review (other)	Varied
273	Waller et al., 2014 [282]	UK	78		Outpatient (adult, females)	To examine whether the efficacy of CBT for bulimic disorders can be translated into routine clinical practice	Case series	Eating behaviours and attitudes, depression pre- and post-treatment, frequency of bingeing and vomiting
274	Watson et al., 2011 [283]	Australia	353		Outpatient (adult, females)	To replicate the model in a sample of women with EDs and to investigate its predictive ability on binge eating and purging	Cross-sectional	Eating Disorder Examination (EDE), Weight Concern and Shape Concern subscales of the EDE (EDE-WSC), Self-Oriented Perfectionism subscale from the Eating Disorder Inventory-2 (EDI-SOP), Rosenberg Self-Esteem Scale (RSES)
275	Watson et al., 2017 [284]	USA	191		Outpatient (adult, both sexes)	To identify predictors and moderators of failure to engage and dropout in both Internet-based and traditional face-to-face CBT for BN	RCT	Failure to engage and drop-out rate
276	Watson et al., 2018 [285]	USA	179		Outpatient (adult, both sexes)	To evaluate the cost-effectiveness of Internet-based CBT for BN (CBT-BN) compared to face-to-face delivery of CBT-BN	RCT	Abstinence from binge-eating and purging; the mean cost per abstinence patient
277	Watson, Fursland and Byrne, 2013 [9]	Australia	972		Outpatient (adult, both sexes)	To describe the prevalence and characteristics associated with early exit at an outpatient ED service	Longitudinal (< 5 years)	Pathology of early exit group compared with non-early exit
278	Wilson and Zandberg, 2012 [286]	USA		136	Outpatient (adult, both sexes)	To determine the effectiveness and scalability of cognitive-behavioural guided self help for EDs	Review (narrative)	Varied
279	Wonderlich et al., 2014 [287]	USA	80		Outpatient (adult, both sexes)	To compare a new psychotherapy for BN, integrative cognitive-affective therapy, with an established treatment, CBT-E	RCT	Bulimic symptoms using generalised estimating equations (GEEs)
280	Zerwas et al., 2017 [288]	USA	179		Outpatient (adult, both sexes)	To compare an Internet-based manualized version of CBT group therapy for BN via a therapeutic chat group to the same treatment via a traditional face-to-face group therapy	RCT	Frequency of binge-eating and purging
281	Zipfel et al., 2014 [289]	Germany	242		Outpatient (adult, both sexes)	To assess the efficacy and safety of focal psychodynamic therapy and CBT-E versus optimised TAU	RCT	Weight gain, measured as increased body-mass index (BMI) at the end of treatment

### Psychotherapies

#### Cognitive behavioural therapy

A total of 30 studies were identified as providing evidence for the efficacy of CBT, the highest number of any psychotherapy in the RR. Of the psychotherapies, CBT has been the most rigorously scrutinised and therefore has the most empirical evidence [162]. Much of the research on CBT has been conducted with individuals with BN and BED, such that CBT utilising a manualised treatment [18] is generally considered the leading psychotherapy for BN (CBT-BN) [162].

Development of CBT has resulted in an enhanced form (CBT-E), delivered either as a version that focuses exclusively on ED psychopathology (CBT-Ef), or a broad form (CBT-Eb) that also targets mood intolerance, perfectionism, self-esteem and interpersonal difficulties [18, 81, 167]. Evidence suggests that CBT may help to reduce binge/purge symptomatology and support regular eating [69, 167]. Furthermore, CBT has been proposed to have clinical utility as a transdiagnostic intervention for multiple diagnoses including AN and BN in general [69, 167]; as well as for more specific AN subtypes such as AN-binge purge (AN-BP), and for EDs in the presence of comorbidities, such as personality disorders [69, 167]. It should be noted that in RCTs examining this type of CBT intervention across ED diagnostic categories, a significantly greater number of sessions are required for CBT for AN compared with CBT for BN. Studies have also generally been based in outpatient settings; and thus, predominantly recruited participants with AN and a BMI at or above 14.5 [163] so it is unclear whether this treatment works across the AN spectrum to include those with a BMI below 14.5.

Several studies have identified the importance of the therapeutic alliance on the effectiveness of CBT on treatment outcomes. Establishing a good therapeutic alliance increased chances of weight recovery for patients with AN, with 80% of the cohort achieving target weight [290]. Strong therapeutic alliance in a group of adolescents with BED was associated with fewer loss of control eating episodes [215]. Additionally, in a group of participants with BN, stronger alliance was related to a greater reduction in bulimic behaviours, regardless of the treatment [integrative cognitive-affective therapy (ICAT) or CBT-E] [21]. In a group of patients with severe and enduring AN, positive therapeutic alliance established later in treatment predicted reductions in ED and depressive symptoms, which was considered to be particularly significant due to the treatment resistant nature of the disorder [248].

Other studies have produced conflicting results; therapeutic alliance in a group of patients with BN was not shown to be associated with treatment outcomes achieved from CBT-E [219]. It was suggested that although therapeutic alliance was strong throughout treatment, it was not a reliable predictor of patient drop-out or reduction in ED symptoms. In another study, therapeutic alliance was unable to predict a reduction in ED symptoms for adults with AN, BN and OSFED [266]. Daniel et al. [71] and Folke et al. [92] have suggested that individual attachment style plays a role in therapeutic alliance in patients with BN, with secure attachment being associated with stronger therapeutic alliance; however, the impact of alliance and attachment on reduction in ED symptomologies was limited.

Another study examining the relationship between therapeutic alliance and weight gain during CBT for AN found that early alliance was not related to subsequent weight gain, albeit early and late weight gain were positively associated with the strength of subsequent alliance, suggesting that weight gain techniques should be focused on early in treatment rather than rely on the therapeutic alliance to action change [45].

##### Anorexia nervosa

In people with AN, CBT has been shown to positively impact emotional regulation, increase set-shifting skills, improve ED pathology, lower global EDE scores, reduce subjective feelings of incompetence and, in combination with nutritional rehabilitation, produce significant weight gain [68, 132, 254, 289]. Despite established evidence of its efficacy, researchers consider a need for more RCTs using larger sample sizes to not only determine the generalisability of effectiveness across a much broader representation of the general population; but to further understand CBTs efficacy in treating different sub-types of the same disorder [162]. One specific area of uncertainty reflects the differences in underlying symptomatology between anorexia nervosa-restricting (AN-R) and anorexia nervosa-binge-eating/purging (AN-BP) subtypes, such that CBT show differential efficacy across subtypes. For example, CBT was delivered to outpatients for 40 weeks, with significant reductions in emotional eating scores for participants with AN-BP and BN post-treatment, but no significant change in those with AN-R; this differential effect was sustained at 6-year follow up [87].

A recent meta-analysis assessing the efficacy of stand-alone psychological interventions for adult outpatients with AN on change in BMI and clinical symptoms, did not find CBT or any of the treatments to outperform TAU [238]. Further, no reliable evidence demonstrated clear superiority or inferiority of a specific treatment, underscoring an urgent need to develop and improve psychotherapies for adults with AN [238]. In an RCT, comparison of CBT-E to active comparators focal psychodynamic therapy (FPT) and optimised TAU found no significant differences in weight gain at post-treatment and 12-month follow-up, although both therapies were more effective than TAU [78]. Zipfel et al. [289] suggested that the equivalent benefit derived from FPT is due to its less directive approach, which is sensitive to difficulties in autonomy experienced by individuals with AN.

In the UK, research into the applicability of outpatient CBT in ‘real-world’ settings for AN and Atypical-Anorexia Nervosa (A-AN), found treatment to be efficacious, with just under half of the study sample achieving full or partial remission, in the short-term, at end of treatment [132]. A systematic review from 2012 conducted on psychotherapeutic treatments for severe and enduring AN highlighted the limited evidence base for CBT as a treatment for AN over the long-term [116]. Research has shown that while specialist ED psychotherapy may have some advantage over TAU, long-term outcomes for AN and BN patients treated with CBT-E is far from adequate, with long-term remission rates far lower than short-term remission [291, 292].

With the recognition of body shape and weight being a core pathological feature of EDs, a phenomenological perspective has emerged which describes a disorder of embodiment [227]. The embodiment disorder has been posited as a combination of apprehension towards the physical form of the body and a loss of experiencing the body from within; instead, drawing on others perceptions to determine how on sees and experiences their own body [227]. A study investigating the role of the embodiment disorder as a possible mediator of CBT-E efficacy on ED symptomatology found that higher presence of embodiment disorder predicted diagnostic instability and mediated the decrease in overall ED psychopathology [227]. These findings highlight the role of disordered embodiment as a maintaining factor and possible contributor to enduring EDs, supporting an integration of CBT-E with the phenomenological perspective, which may serve to increase remission rates.

##### Bulimia nervosa and binge eating disorder

CBT has consistently outperformed active, inactive, and pharmacological treatment trials of BN and BED [162–164]. CBT-BN has demonstrated the ability to decrease binge/purge behaviours by reducing dietary restraint and normalising eating behaviour or reducing weight/shape concern resulting in a decreased desire to diet [167]; it has also been shown to have a positive impact on emotional regulation [180], self-esteem [168] and reduction of negative feelings such as depressive symptoms [114, 152, 164, 168, 254, 290].

A systematic review of CBT for BN and BED included comparisons with active psychotherapy alternatives and demonstrated superiority of CBT in terms of efficacy and long-term effects, when there was adherence to the CBT manual and protocol [162]. Findings reinforce those of an earlier meta-analysis of 53 studies, whereby CBT outperformed other therapies (IPT, group therapy, and FBT) for both BN and BED [114, 239], and of a systematic review of seven CBT-E studies the efficacy of CBT-E for BED as well as transdiagnostic EDs was supported [73].

An outpatient study sought to determine the efficacy of CBT for individuals with BN and Subclinical Bulimia Nervosa (S-BN, classified as OSFED) [282]. Findings supported the contention that CBT is equally efficacious in ‘real-world’ clinical settings as in RCTs [282]. Testing of ‘real-world’ efficacy was also conducted using a larger sample size of individuals with BN and OSFED accessing NHS services with significant improvements observed at post-treatment in terms of global EDE scores [148].

An RCT examining the effectiveness of CBT-BN and psychodynamic therapy (PDT) for adolescents with BN or S-BN found both interventions to be beneficial to patients, with no significant differences in remission rates between intervention groups [242]. Assessment of EDE scores also found binge/purge behaviours to be significantly reduced at post-treatment and 12-month follow up for both groups, with medium to large effect sizes.

Post-treatment binge/purge abstinence rates have been found to be maintained over long-term follow-up periods for CBT-BN compared with other psychotherapies [167]. A meta-analysis of 27 studies found that delivery of CBT resulted in a binge-purge abstinence rate of 42.1% for treatment-completers and 34.6% in an intention-to-treat analysis [167]. At follow-up, mean abstinence rates were 47.3%. Linardon et al. [167] considered these rates to be comparable to results reported by RCTs and therefore findings from RCTs to be applicable in real-world treatment settings. Individuals with more severe binge/purge symptomatology are considered to respond better to CBT and CBT-E through the targeting of self-esteem as a factor for disorder maintenance [283].

A reduction in binge/purge frequency and depressive symptoms in response to CBT at 4 weeks has been associated with a greater chance of remission at discharge among BN patients [261]. However, some studies have indicated that CBT does not improve feelings of shame and self-criticism [95], and it was not able to demonstrate superiority over other psychotherapies for improving self-esteem or reducing depression symptoms in either BN or BED patients [114, 168]. Gale et al. [95] argued that integration of Compassion-Focused Therapy (CFT) into CBT treatment may be effective in targeting these additional aspects of EDs, leading to better outcomes for patients with AN, BN and Eating Disorder Not Otherwise Specified (EDNOS).

A rapid response to treatment is associated with favourable outcomes in individuals with EDs [31, 271, 276], supporting the application of CBT for this cohort, amongst whom it has demonstrated rapid response in a number of trials. For example, a brief CBT intervention was added to routine outpatient ED treatment to facilitate rapid response among women with BN and Purging Disorder (PD) and was highly effective when compared to motivational interviewing (MI); there was no significant difference between MI and TAU [177]. After 4 weeks, 95.6% of patients receiving CBT had responded to treatment compared with 71.4% of patients in the MI group.

Conversely, a comparison of CBT to motivational enhancement therapy (MET), a form of MI, performed in a clinical outpatient setting during a two-phase RCT found no significant difference for patients with BN and OSFED in terms of benefit, treatment adherence, or self-monitoring [144].

A RCT of 154 individuals with BN or OSFED compared efficacy of focused CBT-E (CBT-Ef) to broad CBT-E (CBT-Eb) and found no significant differences between the interventions at post-treatment or 60-week follow-up; showing efficacy in reducing eating pathology scores, though CBT-Eb should be the default treatment due to ease of delivery [18]. However, CBT-Ef was found to be more effective for individuals with less severe Borderline Personality Disorder (BPD), comorbid with BN; while CBT-Eb produced better results in individuals with more severe BPD [262].

Length of treatment may be an influencing factor, with individuals with BED receiving up to 16 weeks of treatment showing the greatest reduction in binge eating symptoms compared to participants receiving up to 8 weeks of treatment [30]. A comparison of CBT-E to IPT for individuals with BN, BED and other EDs found that whilst both groups displayed significant improvements, participants receiving CBT-E had significantly higher rates of remission at 60-week follow up [82]. Both CBT and IPT have been found to be effective transdiagnostically across EDs in the long-term, and individuals who receive CBT tend to have faster response rates to treatment [82].

A meta-analysis of 21 RCTs assessing treatment options for adults with BN suggested individualised CBT, specifically developed for EDs, continues to be the most efficacious treatment in terms of its ability to achieve full remission in patients, followed by guided CBT self-help [236]. Research into the cost-effectiveness of CBT-BN for treatment of adults in Australia, including manualised treatment [18] delivered by a clinical psychologist, was deemed to be 98% cost-effective [156].

Benefits of participating in CBT interventions have been shown to be maintained long-term, as demonstrated in studies at 2 years [214] and 6 years [194] post-treatment. In the study by Poulsen et al. [214], CBT-E for BN was compared to psychoanalytic therapy specifically developed for individuals with BN. At 2-year follow-up, 44% of participants receiving CBT-E had stopped engaging in binge/purge behaviours compared with 15% of participants who received psychoanalytic therapy. Similarly, delivery of CBT to obese individuals with BED resulted in significant remission at 6-year follow-up as their improvement was maintained compared to pre-treatment scores [194]. However, no significant differences were observed between the group receiving CBT and the group receiving behavioural weight loss therapy (BWLT), suggesting equivalent efficacy of both interventions in the treatment of individuals with BED [194]. Comparative advantage of CBT over BWLT at 6-year follow-up was observed for CBT in terms of fewer binge eating episodes and although participants who received the BWLT had a lower BMI at post-treatment, the BMI reduced more in CBT compared to BWLT during follow-up [194].

Despite evidence regarding the superiority of CBT over other available treatments for BN and BED, non-response to treatment in some individuals has prompted the development of novel therapies. An RCT conducted on a sample of women with transdiagnostic binge eating compared traditional CBT for EDs, an appetite focused CBT (CBT-A)—which incorporates strategies for dietary normalisation and food education—and schema therapy, a form of CBT that focuses on early life exposures and their contribution to ED psychopathology [189]. All interventions were able to produce significant reductions in frequency of binge eating; however, those diagnosed with BED rather than BN, showed greater symptom reduction. No significant differences were observed between interventions at post-treatment or follow-up, indicating equivalent efficacy. Treatment of transdiagnostic binge/purge behaviours using CBT-E was also found to be effective in a sample of adolescents, with half of all participants abstaining from any binge/purge behaviours post-treatment and a significant reduction in global EDE scores [262].

Sodersten et al. [237] argue that there has been little improvement in outcomes of CBT over time despite expansion of the intervention to target other emotional aspects of EDs. The authors present the argument that normalisation of eating patterns and behaviours is a critical component of successful CBT and greater focus is required on the behavioural aspects of BN during treatment rather than the emotional and cognitive aspects that appear to be at the forefront of current CBT treatments. Conversely, a study found that people with BN have central coherence and set-shifting inefficiencies which do not differ from those with AN [145]. This suggests that BN patients may benefit from adjunctive approaches, such as cognitive remediation therapy (CRT) to address these inefficiencies [145].

Unsatisfactory outcomes for CBT for some individuals with BN may indicate a need to better tailor treatment to the different personality structures in such individuals. This was assessed in an RCT examining the efficacy of CBT-E against integrative cognitive affective therapy for BN (ICAT-BN) [82]. Results indicated that over-controlled personality types, associated with elevated shyness, self-consciousness and perfectionism, responded more favourably to CBT-E. Patients with an under-controlled personality type, linked to a lack of impulse control, aggression and emotional dysregulation, exhibited lower rates of purging following ICAT-BN [82]. Assessment of mechanisms of action for psychotherapy interventions based on personality traits was investigated by Daniel et al. [71], who compared CBT to Positive Psychotherapy (PPT) and found that attachment styles were strongly associated with binge frequency. However, no significant differences were found between treatment groups for changes in attachment styles or binge frequency at post-treatment.

Additionally, difference in response to CBT has been observed between genders. Analysis of response to CBT by sex in a sex-balanced sample of patients with EDs found that males with BN or OSFED were more likely to achieve remission than females with the same diagnosis. However, males with BN were also more likely to drop out of treatment than females [236].

##### Behavioural weight loss therapy in integrated approaches

Studies have shown that CBT when compared to BWLT can reduce binge eating frequency, but not weight in patients with BED [49, 184, 194]. Pacanowski et al. [199] found some BED patients gained weight when treated with CBT despite decreases in psychological ED symptomatology. Other studies have been unable to demonstrate any significant differences between CBT and BWLT groups in treatment outcomes [109, 194, 200].

Mason et al. [185] conducted an RCT comparing CBT to a waitlist control for patients with BED. It was found that whilst CBT was effective in targeting psychosocial aspects and ED symptomatology, integration of exercise therapy led to improved weight loss and subsequent improvement in weight-related quality of life (QoL) [185]. Integration of physical activity and CBT interventions to treatment of patients with BED was supported by evidence from a systematic review which indicated that exercise may decrease food cravings and negative affect, increasing the efficiency of CBT treatment [37].

A recent RCT compared the efficacy of introducing a BWLT—the Health Approach to Weight Management and Food in Eating Disorders (HAPIFED)—with CBT-E for the treatment of individuals with recurrent binge eating and BMI ≥ 27 kg/m^2^ [200]. A significant reduction in ED symptoms was observed in both the HAPIFED and CBT-E groups and was sustained at 12-month follow-up. No differences between groups were observed for weight loss; however, a greater reduction in purging behaviours and binge remission rates at 12-month follow-up were demonstrated with HAPIFED.

##### Avoidant/restrictive food intake disorder

As a recently characterised disorder, ARFID has significantly less research than other EDs presented in this RR. Research on CBT as a treatment for ARFID is limited to case series, case reports and retrospective chart reviews, with only a few RCTs conducted on young children [258]. A protocol paper by Thomas and colleagues, describes the development of a modified CBT for ARFID in children, adolescents and adults, and an open trial which compares the therapy to FBT approaches [257].

A pilot trial titled “Supportive Parenting for Anxious Childhood Emotions adapted for ARFID” (SPACE-ARFID)[235] evaluated program outcomes related to a parent-based intervention for ARFID, which focusses on parent response to problematic eating and aims to increase food flexibility. Fifteen families of young people with ARFID aged 6–14 years old were recruited into the program. There was a 93% completion rate, high satisfactory rating of treatment, significant reductions in symptom severity, impairment and family accommodation, representing promising preliminary evidence for feasibility, acceptability, and potential for improvement in outcomes [235].

##### Other specified feeding or eating disorder

Although there is evidence to suggest that CBT may be an effective treatment for the full range of EDs due to its targeting of transdiagnostic symptomatologies, it is consistently under-researched for ‘non-core’ disorders [18, 204]. Only one publication was identified by the RR assessing the effectiveness of group CBT in a sample population of individuals with OSFED [204]. No studies were identified that addressed CBT treatment for Night Eating Syndrome (NED). In a longitudinal study of adolescents, Thompson-Brenner et al. [260] suggested a potential treatment gap for OSFED, as patients with this diagnosis were less likely to receive CBT than patients with AN or BN.

#### Family based therapy

Evidence for the effectiveness of FBT currently exists primarily for the treatment of adolescents with AN. Trials have been expanding and include protocols for new populations and diagnoses, including BN, with clinical guidelines now recommending an ED-specific FBT as the first-line treatment for adolescents with AN, and as a recommended intervention for adolescents with BN [100].

Four publications were identified relating to BN [59, 67, 196, 267], while no studies of FBT for the treatment of BED or ARFID were identified. Loeb et al. [172] argued that FBT’s targeting of family issues, including blame, internalisation of illness and parental response to the ED, suggests transdiagnostic utility, but further research in samples with diagnoses other than AN are required to evaluate this.

Researchers assessed studies on FBT (FT-AN and FT-BN) including multi-family therapy (MFT) and group therapy involving several families of individuals with AN or BN, finding that successful treatments: (1) use the family as a resource to promote changes in ED behaviours and symptomatology early on in treatment; (2) are delivered by clinicians with expertise in EDs, preferably within a multidisciplinary team; (3) are delivered in a consistent manner, ideally adhering to treatment manuals but remaining flexible enough to meet the individual needs of families; and (4) develop a therapeutic alliance with adolescents and parents, facilitating learning and behaviour modification [133].

Evidence from a systematic review of six RCTs assessing the effectiveness of FBT for adolescents with AN, BN and OSFED found that whilst no significant post-treatment differences were observed between FBT groups and those receiving individual treatment, FBT was superior at sustaining treatment effects for all EDs at 6 and 12-month follow-up [67].

A number of variations to the FBT protocol have been developed and have demonstrated varying treatment outcomes. Whilst most trials of adaptations to the traditional FBT format reported favourable outcomes for patients, Richards et al. found that very few of these studies used FBT as an active comparator; thus, limiting support for the efficacy of FBT protocol augmentations [222].

##### Anorexia nervosa

Guidelines indicate FBT is the leading treatment for AN, and suggest its use for the treatment of adolescents with other EDs [65, 67]. FBT has demonstrated effectiveness in reducing symptomatology following treatment for AN, sustained [65, 196, 293] at 6 and 12-month follow-up [171]. Decisive parental action and involvement early on in treatment have been associated with positive outcomes for restrictive type EDs [181]. Rapid response to treatment has been found to be strongly associated with a positive prognosis, with early weight gain predicting greater weight gain and remission at the end of treatment, as well as improvements in other ED measures [20].

Involvement of parents and siblings in FBT has been found to be associated with better outcomes, with results indicating that sustained involvement of fathers over a 6-month period was associated with higher rates of remission [65, 130]. To try to prevent poor outcomes in adolescents who did not have a rapid response to treatment following their first four sessions, Lock et al. [171] developed a novel adaptation to standard FBT. This treatment provides intensive coaching to parents on self-efficacy and refeeding, resulting in weight restoration at post-treatment comparable to rapid responders [171]. However, findings from this study are limited by a small sample size (*n* = 45), particularly the comparator group (*n* = 10) [171]. Whilst greater parental self-efficacy is a known predictor of favourable FBT outcomes, further research is required to understand the impact of intensive parental coaching on treatment response [225].

A variant of FBT, parent-focused therapy (PFT) in which sessions involve primarily parents with limited contact between the therapist and adolescent, was found by Le Grange et al. [160] to produce rapid response in adolescents with AN. At post-treatment, adolescents in the PFT group were three times more likely to achieve remission than those receiving FBT. These differences were no longer statistically significant between groups at 6- and 12-month follow-up despite higher remission rates for PFT compared to FBT. PFT therefore represents a viable alternative to FBT that can produce rapid response in adolescents with AN. However, findings indicate that it is not more effective than FBT in the longer-term [160].

Systemic family therapy (SyFT), a further variant of FBT, has a focus on the relationships and interactions present within the family system [22]. Unlike FBT, there is no family meal component or emphasis on normalisation of eating. Results from a randomised parallel study indicated no significant differences in outcomes between FBT and SyFT, suggesting they are equally effective. However, researchers noted that SyFT may be more effective in adolescents with more obsessive–compulsive symptomatology or comorbidity, known to predict lower response to treatment [22].

Increasing the effectiveness of FBT treatment through additional support provided by a network of families with children or adolescents undergoing treatment for AN is explored through multi-family therapy (MFT). In an RCT comparing FBT-AN to MFT-AN, benefits were observed in both groups at 18-month follow-up, with a significant difference in increases to BMI for the MFT-AN group [79].

A meta-analysis including 19 RCTs synthesised the best available evidence of non-pharmacological face-to-face interventions on BMI, body dissatisfaction, depression and anxiety among AN individuals [94]. Behavioural family system therapy (BFST) was found to be more effective than ego-oriented individual therapy in enhancing BMI, and conjoint family therapy (CFT) more effective than separated family therapy in reducing depression, with combined family and individual therapy producing larger effect sizes than individual therapy alone [94]. Conversely, results from RCTs consider FBT and adolescent-focused individual therapy (AFT) to be equally beneficial in terms of clinical outcomes relating to weight gain, reduction of ED behaviours, and depressive symptomatology [94, 130]. Based on results from a multi-site RCT, Le Grange et al. [158] argued that FBT is more effective than AFT for AN patients with more severe symptomatology. Moreover, FBT has been demonstrated as a cost-effective approach to treating adolescents with AN [20, 157].

A comparative analysis of psychotherapy modalities by Brauhardt et al. [40] found that the evidence supports the effectiveness of FBT over individual therapy for adolescents with EDs, but not adults. Similarly, a recent Cochrane Review [88] of FBT for AN included 25 trials in adults as well as adolescents and was also unable to find any significant benefit of the approach over other therapies, particularly in terms of achieved remission or weight gain [88].

ED drop-out rates are a significant issue, with up to 50% of inpatients and 57% of outpatients being treated for AN not completing FBT [102]. A systematic review and meta-analysis of 27 studies found that factors which predicted family therapy treatment drop-out in adolescents with AN included having AN-BP, low BMI, and low motivation, which should be assessed at commencement and actively addressed over the course of treatment [40]. These high dropout rates may reflect expert consensus that family therapies such as FBT are only suitable for some families and some presentations, and highlight the need to develop a number of alternate evidence-based therapies suitable for children and adolescents with AN.

##### Bulimia nervosa and binge eating disorder

Studies exploring BN-focused FBT are limited, and no studies investigating FBT for BED were identified as part of this RR. In an RCT comparing FBT-BN to CBT for adolescents (CBT-A), FBT-BN was found to be significantly superior to CBT-A at post-treatment and 6-month follow-up in terms of reducing bulimic symptomatology, with a significant proportion of the FBT-BN group abstinent from bingeing/purging compared with the CBT-A group [159]. There were, however, no significant differences at 12-month follow-up. These results indicate that FBT-BN has the capacity to produce a rapid response in adolescents with BN and it has been suggested that it may be a better alternative for individuals with more severe bulimic symptomatology [159].

Investigations into the impact of FBT for comorbid depression and self-esteem in the same sample population was published in a subsequent study [267]. Results indicated FBT-BN and CBT-A to be equally effective at reducing depressive symptomatology and increasing self-esteem in adolescents with BN without any significant differences between treatment groups at post-treatment or follow-up. Of note, depressive symptomatology was found to decrease in both treatment groups between post-treatment and 12-month follow-up [267]. These studies indicate that FBT-BN is a viable alternative to CBT for the treatment of BN in adolescents.

In an RCT, FBT was compared to individual focused supportive psychotherapy for patients with either BN or S-BN; the interventions were found to be equally efficacious in reducing ED symptoms including binge/purge frequency at end of treatment and 6-month follow-up [59].

A small study investigating an integrated FBT-DBT approach found promising results [274]. FBT-DBT targets emotional regulation aspects of BN and this is thought to mediate reduction in binge/purge symptomatology [274]. Although the intervention was able to produce significant reductions in bulimic symptomatology, it is a comprehensive and intensive treatment potentially only required in severe and complex cases. Findings are also limited by the small sample size, absence of a control group and follow-up data [274].

##### Avoidant restrictive feeding intake disorder

No published studies were identified that assessed FBT treatment for ARFID; however, several trials are currently being conducted [259]. FBT for ARFID is considered to work on the same premise as FBT-AN. In addition to the parent encouraging the child or adolescent to increase the volume of food consumed, a focus is also placed on the variety of foods eaten [259].

##### Other specified feeding eating disorders

Two studies [252, 293] were identified with a focus on FBT for adolescents with A-AN and restricting type ED symptomatology that were considered to be classified as either other OSFED or UFED. Results from these studies indicated that benefits for adolescents undergoing FBT were similar for both AN and A-AN. This is despite the primary goal of FBT being weight gain, with up to 52% remission and a significant reduction in EDE scores and purging behaviours post-treatment[293], and up to 53% remission and a significant reduction in EDE scores at 12-month follow-up [252].

#### Dialectical behavioural therapy

Originally developed to treat Borderline Personality Disorder (BPD), particularly symptoms relating to suicidality and self-harming behaviour [294], DBT has been adapted for the treatment of EDs with some success, in particular in the EDs with bingeing as core symptomatology. However, RCTs, particularly large RCTs, investigating the effectiveness of DBT are few [162] and some such studies have been uncontrolled [32].

Studies focusing on DBT and DBT adapted for BED (DBT-BED) have indicated that this approach may be successful in reducing the frequency of binge-eating [58, 217]. Sixty women with BED were provided 20 2-h sessions of DBT across 10 weeks, and results post-treatment showed a significant reduction in BMI and binge eating occurrences, as well as improvements to emotional regulation [217] Similarly, a study of 109 women with BN and BED were administered weekly DBT sessions over a 6-month period and improvements in ED symptomatology were observed for all participants at post-treatment. At 12-month follow-up those treated with DBT had significantly less objective binge eating than those in CBT guided self-help (CBTgsh) group [58].

Results from an open trial of DBT involving women with comorbid ED (AN and BN) and BPD who did not respond to previous, non-DBT oriented treatment, resulted in a remission rate of 54% in BN (fewer binge-eating episodes) and 33% with AN (increased weight) at 15-month follow-up; however, the study’s definition of ‘remission’ was not made clear [150]. Further, it should be noted that 44% of individuals with AN converted to BN at follow-up [150].

Development of an adapted DBT for BN was explored in a study which incorporated appetite awareness training (AAT) with DBT to produce appetite-focused DBT (DBT-AF) [171]. Compared to CBT—which monitors volume and type of food, AAT seeks to increase awareness of internal appetite signals [124]. In this study involving 32 women with BN and S-BN receiving DBT-AF for 12 weeks, positive results were observed at post-treatment, with 61% of participants achieving full remission, and 27% binge/purge abstinence compared to waitlist controls who reported higher objective binges/purges and higher scores on EDE-Q. However, the researchers draw specific attention to the limited sample size and suggest findings should be interpreted with caution [124].

#### Interpersonal therapy

Considered a less directive approach to therapy than CBT, IPT focuses on interpersonal problems and their relationship to psychological symptoms [295]. The IPT-ED model seeks to help the individual establish a sense of social acceptance that negates the need to engage in ED behaviours [223]. A small number of studies were identified relating to IPT for EDs. IPT is more commonly mentioned in the literature for EDs as an active comparator to CBT interventions. While this psychotherapy is considered a viable and effective treatment for EDs, many researchers consider CBT superior than IPT in producing rapid response, which may have led to a lack of recent research investigating this intervention [167].

In a recent systematic review on IPT for EDs, IPT was shown to be the least effective for AN in respect to overall global scores when compared with CBT and nonspecific SSCM at post-treatment [295]. However, at 5-year follow-up, those initially randomised to IPT demonstrated the greatest global outcome rating, with a “lag” effect occuring over time, with only 29% of the followed-up sample (n = 56) having accessed additional ED support in this period [296]. Similarly, following treatment, IPT was less effective than CBT-BN and behavioural therapy (BT) for reducing BN symptoms [295]. However, when participants were evaluated at 8-month follow-up, the IPT group showed further improvements [295]. Comparatively, BT was least effective overall with significant post-treatment relapse rates [295]. At 6-year follow-up, IPT and CBT-BN demonstrated equivalent and enduring reductions in general psychiatric features and improved social functioning/self-esteem [295, 296]. The results suggest efficacy for CBT-BN and IPT over the intermediate term (months), and particular advantages for IPT over the longer term (years).

A RCT investigated the efficacy of IPT in producing a rapid response in adults with BED, against two active comparators: BWLT and CBTgsh [122]. IPT was found to be the least effective of the interventions with 65% of participants showing a rapid response rate compared with 74% for CBTgsh and 73% for BWLT [122]. Rapid response in the CBTgsh group was found to predict greater remission in binge eating. However, this effect was not observed in the IPT or BWLT groups. These results indicate that although IPT is not effective in generating a rapid response to treatment in adults with BED, this has limited impact on its effectiveness in producing symptom remission [122].

A review of IPT-ED confirmed that while it can be recommended for BED and BN, IPT could not be recommended as a treatment for AN and identified a gap in the evidence regarding use of IPT for EDs not otherwise specified [195].

#### Acceptance and commitment therapy and integrative cognitive affective therapy

Juarascio et al. [136] presented the argument that ACT may be more effective than CBT at targeting key features contributing to ED maintenance, including experiential avoidance, awareness and lack of motivation to change, which are particularly relevant for individuals with AN. To test this proposed mechanism for behaviour change, Juarascio et al. [139] conducted an RCT in a sample of college students with diagnosed EDs or sub-clinical ED symptomatology, comparing ACT to CBT. While results from the trial indicated that ACT was more effective than CBT at reducing ED symptomatology, the study is limited by its delivery of non-manualised therapy and the very small number of individuals (*n* = 55) with a diagnosed disorder.

A subsequent RCT of ACT, in an inpatient ED setting had a more rigorous methodology, employed a TAU control, and included follow-up assessments [202]. This study was unable to demonstrate an advantage for ACT over TAU in reducing ED symptoms. While the intervention had a moderate effect on symptomatology, differences between groups did not reach statistical significance [202]. An additional comparison of ACT to TAU in an RCT was also unable to demonstrate the superiority of ACT over the control condition [202].

An RCT conducted by Wonderlich et al. assessed the efficacy of Integrative Cognitive-Affective Therapy (ICAT) for BN, delivered over 19 weeks [287]. ICAT proved to be as effective as CBT-E in producing a moderate to large effect on bulimic symptomatology, with no significant differences between groups as measured at post-treatment or follow-up. This finding was supported in a subsequent study by Peterson et al. where the treatment period was extended to 21 weeks [208].

Recognising the need for innovative treatments for BED, an RCT compared ICAT-BED with CBTgsh [209]. Binge eating frequency showed significant reductions post-treatment and at 6-month follow up, with no differences between therapy groups [209]. Maintenance mechanisms and measures of associated ED psychopathology, negative affect, depression and anxiety showed improvement post-treatment and at follow-up, with no differences between therapies. Treatment retention was significantly higher for ICAT-BED participants than for CBTgsh.

A subsequent RCT examined predictors and moderators of frequency of binge episodes associated with ICAT and CBTgsh for BED, finding the predictor actual-ideal self-discrepancy interacted with the treatment type to differentially predict binge episode frequency post-treatment and at 6-month follow-up [29]. Thus, ICAT-BED may produce specific and long-lasting improvements in binge frequencies among those with high actual-ideal self-discrepancy.

#### Exposure-based therapies

Exposure-based therapies work to establish, through experiential learning, that negative consequences do not occur when the individual is exposed to a feared stimulus [245]. These are not standalone treatments but often used in conjunction with other interventions, most commonly CBT, to increase the effectiveness of treatment and target aspects not covered by routine psychotherapy.

A recent systematic review on exposure interventions for AN, BN and BED included 60 studies on exposure and response prevention (ERP), mirror exposure, in vivo feared food exposure, family-based treatment with exposure, and virtual reality exposure therapy [47]. In vivo exposure to feared foods was found to increase caloric intake and BMI, and reduce state anxiety, although the research was limited. Likewise, mirror exposure regardless if conducted in the context of CBT treatment, was found to reduce body dissatisfaction and marginally improve binge/purge cues when treated using ERP compared with CBT [47]. However, a majority of these conclusions were based on lower-quality RCTs (n < 50), limiting their generalisability. In a separate study, Diaz-Ferrer et al. assessed the effectiveness of mirror exposure in patients with BN [75]. Results indicated that both guided and unguided or ‘pure’ mirror exposure had clinical utility for this group for reducing body dissatisfaction [75].

Evidence from other reviews have indicated that mirror and body exposure techniques in combination with CBT can help restructure negative beliefs in individuals with EDs [104, 245, 253]. A small RCT comparing ERP to Cognitive Remediation Therapy (CRT) in an inpatient group with AN found that ERP was significantly more effective at increasing participant food intake). A systematic review investigated the application of ERP strategies into treatment protocols and their effectiveness for binge eating [179]. Around 50% of those receiving CBT for binge eating fail to recover, identifying the need for this investigation to extend into additional exposure strategies. ERP was found to effectively reduce binge eating episodes in the included studies, with the effect sizes largest in samples with the most severe binge-eating behaviours.

Moreover, an ≥ 80% reduction in binge frequency occurred between pre- and post-treatment in studies utilising all relevant food cues, in-vivo exposure, occasional reinforcement and exposure practiced in multiple contexts [179]. Results suggested that ERP is more effective than self-control techniques and CBT without ERP, in reducing binge eating in the long term. This outcome was not evident in the short-term, suggesting that ERP may be effective when other treatments have not been successful [179], or may be reflective of longer-term trajectory of outcome of the treatment. Although in-vivo is the recommended method of exposure, ERP delivered through virtual reality (VR) demonstrated a reduction in binge eating by 90%, suggesting this as an effective alternative. However, more research is required comparing these forms of delivery [179].

An RCT sought to evaluate ERP for AN (AN-EXRP) as compared to Cognitive Remediation Therapy (CRT) for hospitalised patients who had restored weight to BMI > 18.5 kg/m^2^ with treatment outcome assessed by change in caloric intake [246]. Those receiving 12 sessions of AN-EXRP increased average test meal caloric intake from 352 ± 263 kcal to 401 ± 215 kcal post-treatment, while those who received CRT decreased from 501 ± 232 kcal at baseline to 424 ± 221 kcal post-treatment. This increase was significantly associated with improvement in eating-related anxiety.

A 5-year three-arm RCT explored the efficacy of an ERP intervention following treatment with CBT for 135 individuals with BN [188]. The intervention involved two variants of ERP: ERP-pre-binge and ERP-pre-purge; the third group received relaxation therapy following CBT treatment. At 5-year follow-up, both ERP treatments were significantly more effective at producing abstinence from bingeing behaviours compared to relaxation therapy. ERP was also able to reduce purging behaviours, although no significant differences were noted in abstinence rates for this behaviour. This indicates ERP is a useful add-on to routine CBT for BN patients to reduce long-term symptomatology.

The medication D-cycloserine, which has a role in neurotransmission, was thought by Levinson et al. [161] to enhance the benefits of ERP by increasing facilitated experiential learning. A trial supported the efficacy of D-cycloserine, with the active group receiving exposure therapy and pharmacotherapy gaining significantly more weight than the placebo group, who received therapy alone. It should, however, be noted that most people in this study were not underweight upon entering, with 81% having a BMI ≥ 18.5 [161].

The efficacy of approach bias modification (ABM) training as a novel intervention to reduce binge eating in patients with BN and BED has been investigated in an RCT [42]. Participants in the active group were taught to avoid food cues as a means of reducing reactivity to food in subsequent exposures. Results from the trial indicated a significant reduction in objective binge eating episodes with no difference between the active and ‘sham’^1^ control group. However, larger reductions in other ED symptoms were observed in the active group compared to controls [42].

#### Other psychotherapy approaches

Several other psychotherapy approaches to treating EDs were identified in the RR. These included mindfulness, emotionally focused therapy, self-compassion, self-identification, motivational interviewing (MI), and body movement and body awareness therapies. With the exception of the systematic reviews on mindfulness-based therapies and MI (see below), much of the evidence presented is preliminary and from studies with small sample sizes and should be considered as requiring further examination of their potential use in EDs. Specifically, a cross-sectional study of Australians with diagnosed and undiagnosed EDs (*n* = 425) suggested that having more widely available and novel interventions for EDs would more likely encourage people to seek help [106].

Evidence from two reviews on mindfulness-based therapies [143, 198] support its capacity to reduce binge eating and emotional eating behaviours in individuals with obesity and BED. Approximately 90% of the mindfulness interventions reviewed by O’Reilly et al. [198] resulted in modest weight loss in participants. Similarly, Katterman et al. [143] reported moderate to large effects on measured binge eating frequency and superiority to BWLT, with no significant differences measured between treatment outcomes from mindfulness training and other active comparators, DBT and IPT.

There is evidence of efficacy for blended mindfulness, compassion and psychoeducation interventions in reducing ED and depressive symptoms in patients with BED compared with waitlist controls [211]. Also, compassion-focused therapies for individuals with BED showed a reduction in weekly binge days and eating and weight concerns compared to a control group [146]. An intervention incorporating aspects of CBT, IPT and DBT treating emotional dysregulation with BED or S-BED achieved significant reductions in ED symptoms as measured by the EDE-Q compared to waitlist controls, and were maintained at 12-month follow-up [60]. However, given the small sample size and a lack of supporting data from other studies using the same approach, further research is required to determine the clinical utility of this intervention. The efficacy of an emotional and social mind training program was assessed against group CBT for patients with BN, with global EDE scores not significantly different across treatment conditions [154].

A novel identity-based intervention was examined in an RCT involving 69 women with AN, A-AN, BN or S-BN [243]. The intervention was developed to target disturbances in self-concept and negative self-schemas common across these diagnoses [243]. The intervention was found to produce a significant reduction in drive for thinness in comparison with a supportive psychotherapy control group. However, no significant differences were observed between groups for other ED symptomologies.

Two RCTs conducted by Boerhaut et al. [38, 39] investigated a novel approach based on psychomotor therapies (brief body and movement-oriented intervention) used in other mental health interventions, such as the treatment of posttraumatic stress disorder. Both studies found significant reductions in ED symptomatology in the intervention group compared with TAU [38, 39]. Another study on body awareness-based strategies for EDs found that it reduced drive for thinness and body dissatisfaction in outpatients with EDs in comparison with outpatient controls [54].

Evidence from a systematic review of 13 studies assessing the use of MI approaches in ED treatment indicated that these interventions were effective at increasing ‘readiness to change’ among study participants [176]. Considering that resistance to treatment is common among individuals with EDs, incorporation of MI may work to increase initial treatment uptake and continued long-term improvement [77, 176]. A review of the effectiveness of motivational interviewing in the treatment of EDs did not find any significant benefits compared to TAU, although increased motivation to change was observed in participants with BED and BN [77].

### Psychotherapy delivery modalities

#### Self-help

Self-help interventions can be delivered with therapist input, guided self-help (GSH), or independently—‘pure’ self-help. All studies identified describing self-help interventions targeted individuals with BN and BED or binge/purge OSFED subtypes. Recent clinical guidelines have recommended GSH as the first-line treatment for non-underweight EDs characterised by recurrent binge eating, namely BED and BN [34, 113]. Evidence for its efficacy in individuals with BED is particularly strong [53, 251, 265, 286]. Self-help interventions are not recommended in the treatment of AN due to the specialist care required for this disorder [286].

GSH is often briefer than traditional clinician-led therapy, and has been specifically developed to be used in non-specialist settings [46]. It has been suggested that the comparative advantages of self-help over therapist-led interventions include increased access, cost-effectiveness and capacity to be delivered by professionals without specialist knowledge in ED treatment [175]. However, an economic analysis of individual CBT compared to CBTgsh found only a non-significant difference in cost-effectiveness between the interventions, with both interventions having an estimated cost under the willingness to pay threshold of approximately $440 AUD per binge free day used in the study [149]. While individual CBT was associated with a slightly greater cost, it also resulted in a higher number of binge free days [149].

An RCT investigated the effectiveness and cost effectiveness of GSH via face-to-face delivery (fGSH), and a more scalable method, providing email support (eGSH) for adults with BED [131]. The primary outcome was improvement in ED psychopathology, and for cost-effectiveness, binge-free days. Both forms of GSH were superior to the waitlist control group in reducing psychopathology and binge eating, and both were cost-effective compared to control [131].

A systematic review and meta-analysis of self-help for BN and BED concluded that professional guidance significantly improved the efficacy of such interventions, with benefits derived primarily from increased program adherence and clinician specialisation in EDs resulting in higher treatment completion rates and larger effect sizes [34]. Evidence from meta-analyses and systematic reviews has found that therapist-led CBT achieves better results for individuals with BED [46].

In settings where there are access issues, CBTgsh can be offered as a first-line treatment [46, 273]. Some RCTs conducted using guided self-help based on CBTgsh have not found any significant differences when compared with full therapist-led individual CBT interventions [286]. ‘Pure’ self-help was not found to be effective compared to usual care in a study of obese individuals with BED [108]. The population involved in this study were from Culturally and Linguistically Diverse (CALD backgrounds, which may have had an impact on the findings. There is evidence to suggest that CBTgsh has demonstrated benefit within the CALD population who may benefit from additional culturally appropriate guidance [286].

Assessment of the efficacy of internet-based CBTgsh compared with traditional workbook CBTgsh found both interventions to be equally effective at reducing BN symptoms [279]. An earlier study of 155 women with BN produced similar findings regarding the comparative effectiveness of internet CBTgsh versus bibliotherapy [278]. Similarly, an RCT found that a digitally delivered CBT self-help intervention for binge-purge EDs was effective in decreasing overall ED psychopathology, compensatory behaviours, depression and clinical impairment compared to controls [89]. A study into attitudes toward online guided self-help among individuals with BN found that it was highly acceptable and identified weekly email, text message or forum support as the most preferred forms [187]. Comparison of CBTgsh with email support from therapists to an intensive outpatient program for BN and S-BN found that, although larger effect sizes were observed in the group receiving intensive face-to-face treatment, both interventions significantly reduced bulimic symptoms [126].

Self-help interventions targeting transdiagnostic binge/purge behaviours common to BN, BED and several OSFEDs have reported no significant differences in efficacy across diagnostic groups [218, 251, 265, 270, 271]. Higher drop-out rates have been observed in individuals with BN compared to BED, with findings of a systematic review and meta-regression suggesting that clinician-guidance during self-help treatment for BN may improve both treatment adherence and outcome [34]. Depending on diagnosis, differences in motivation to persist with self-help interventions may be related to change in weight [34]. Specifically, participants associated BED treatment with weight loss, whereas BN treatment was associated with weight gain [34]. A study investigating potential factors that may contribute to completion of treatment using GSH for BED found that non-completers experienced higher pre-treatment levels of weight concern and depression, and lower levels of general health and energy, with main reasons for discontinuing related to (1) perceptions of the GSH program; (2) program practicalities; and (3) the individuals readiness to change [135].

Investigation into predictors of outcome following CBTgsh for BN identified a need for greater focus on relapse prevention as part of the intervention. Results from this RCT of 87 individuals with BN found that increased global EDE scores at 6-month follow-up were associated with lower levels of binge eating, stress and positive thoughts about eating at post-treatment [240]. Further, it has been suggested that targeting perfectionism and motivation to change early on in ED treatment could lead to improved outcomes for individuals with BN and enhancing an early response to treatment has emerged as the most significant predictor of binge eating remission [240, 271].

Although GSH is not a recommended treatment for AN, it has clinical utility for this group as a tool for increasing engagement in routine treatment. Brewin and colleagues examined the effectiveness of Motivation and Psycho-educational guided self-help intervention for people with Eating Disorders (MOPED), an MI-based approach [41]. MOPED was unable to increase completion rates in patients with BN spectrum disorders (62%) compared to TAU (51%). However, among AN patients the intervention was highly effective, resulting in 63% of those receiving the GSH-MOPED intervention completing routine treatment compared with 29% of the TAU group. Another RCT assessing the utility of providing MI in addition to ‘pure’ self-help resulted in greater readiness to change and self-efficacy compared to the group receiving psychoeducation and self-help [272]. Both interventions were found to reduce binge eating in the study population.

#### Group therapies

The majority of group-based studies in this RR were in people with BED, indicating that group therapies may be more effective or acceptable for individuals with this disorder. Several studies were conducted on BED, BN, and subthreshold BED (e.g., OSFED) group treatment, with some promising results [221, 247].

Research findings are consistent regarding the effectiveness of CBT for reducing binge/purge behaviours in BN and BED with a considerable body of evidence supporting its delivery in a group format [24, 66, 134, 233]. Aguera et al. [24] noted that CBT delivered in a group format was considerably more effective for individuals with BED than those with BN, with 70% of BED participants achieving full remission compared with between 31 and 36% in the BN groups.

A study making direct comparison of individual and group CBT in patients with BED found that, while only individual CBT was able to reduce core ED symptoms such as weight/shape concern, both modalities achieved significant reductions in binge eating frequency, with similar rates of remission at 3-year follow-up. Variation in the length of active group CBT treatment has been found to contribute to remission rates. At 125-month follow-up > 99% of individuals receiving 16 sessions (CBT-L) achieving full remission compared with 64% of those receiving 8 sessions (CBT-S) [233].

In an analysis of the impact of group dynamics on treatment outcomes between therapist-led, therapist-assisted, GSH and group CBT, in a sample of individuals with BED, benefit was found for all intervention types, with no association between group dynamic and reduction in ED symptoms [212]. A separate analysis of the impact of group settings found benefit, specifically showing that participation in group IPT improved self-esteem for women with BED by reducing negative self-view through the feedback offered by other participants [96].

Meta-analyses investigating group therapies for BN have not found sufficient evidence to support the theory that group CBT has a clinical advantage over individual CBT or other therapies [213]. Research findings are consistent regarding the effectiveness of CBT for transdiagnostic binge/purge behaviours in BN and BED with a considerable body of evidence regarding its delivery in a group format [24, 66]. A CBT group intervention with incorporated physical activity counselling resulted in significantly increased physical activity and Health-Related Quality of Life (HRQoL) in participants, as well as significant reduction in binge eating frequency and BMI [269]. These results are encouraging, especially in light of HRQoL in obese patients with BED being consistently worse than in obese controls without BED and non-obese individuals [268]. However, the study did not include a control group, and large-scale RCTs are required.

In a study of patients with OSFED, Riesco et al. [224] assessed the effectiveness of group CBT and the potential impact of clinical heterogeneity on outcomes in women with A-AN, PD and S-BN. Participants were assigned to groups with a mix of diagnoses. Between 10 and 22% of participants achieved full remission following the intervention with no significant difference between diagnostic groups; drop-out rates were also similar across diagnostic groups. While these findings indicate that CBT may have clinical utility across OSFED diagnoses, full remission achieved by participants in the study was considered low. Riesco et al. [224] argued this may be attributable to low motivation to change, typical across OSFEDs. Therefore, treatment for OSFEDs may require additional features to address this issue.

Studies investigating psychotherapies other than CBT delivered to groups have shown mixed results for people with EDs [147]. Non-CBT interventions that were found to have benefit included IPT, emotionally-focused group therapy (EFGT) for individuals with BED, CD-Rom group therapy, and self-esteem and social skills therapy for adolescents with AN and BN. Deficiencies in social and interpersonal skills are highly associated with having an ED within clinical samples [219].

Group IPT for BED has emerged more recently than group CBT and is empirically associated with the management of negative emotions as dysfunctional responses to interpersonal stressors [295]. A recent systematic review included a study comparing the efficacy of group CBT, group IPT and waitlist on non-purging BN and found significant reductions in binge eating at post-treatment for CBT and IPT groups, which remained below baseline at 6-month and 1-year follow-up [295]. Similarly, group CBT and group IPT produced similar outcomes for patient recovery rates post-treatment (79% for CBT vs 73% for IPT) and at 1-year follow-up (59% for CBT vs 62% for IPT), with the frequency of binge eating remaining below baseline [223]. At 4-year follow-up, a significant and long-lasting benefit for CBT and IPT was shown, and a comparable remission rate to a subclinical level of BED [122]. For IPT patients, abstinence from binge eating remained stable and reduction of psychopathology was maintained or improved, whereas CBT patients had a significant tendency to relapse and psychopathology worsened during 1-year and 4-year follow-up compared to those treated with IPT.

Two studies found EFTG and EFTG in combination with dietary counselling to be highly effective in reducing ED symptomatology and weight in individuals with BED [61, 62]. Dissonance-based group therapy was compared to a supportive mindfulness group intervention for women with a diagnosed ED and no differences were found in ED symptoms between groups; however, the dissonance-based treatment group demonstrated a higher rate of remission than the supported mindfulness group [247].

In a child and adolescent population study, social skills group therapy and self-esteem group therapy were effective for individuals with AN-type disorders and BN-type disorders, with no significant differences between groups on measures of self-esteem [155]. Social skills group therapy was more effective in the BN-type group. Emerging evidence into the delivery of group-oriented FBT appears to be yielding promising results particularly as a support network for families with children in treatment, with MFT-AN leading to significant increases in BMI compared with individual FBT [79].

#### Technology-based interventions

The largest proportion of studies in the RR investigating technology-based interventions relate to the online delivery of CBT (iCBT). iCBT interventions have been found to result in significant reductions in ED symptoms compared with controls among individuals with BN, BED and OSFED [255, 278]. iCBT produced significant improvements in ED symptoms among participants with BN and S-BN [52], and the efficacy of iCBT for participants with BN and OSFED has also been supported [229, 250]. An RCT of iCBT with GSH for BED found that while it was inferior to individual face-to-face CBT in reduction of binge eating at 4-month follow-up, there were no differences between the online and face-to-face interventions at 18-month follow-up [250]. This suggests that iCBT with GSH is a viable alternative to face-to-face therapy, in addition to being easier to access and more affordable.

A systematic review of five studies on iCBT for BN, S-BN and OSFED concluded that iCBT has the capacity to reduce binge/purge behaviours but has not demonstrated superiority over bibliotherapies or waitlist conditions [297]. This finding reinforced an earlier systematic review of 21 computer-based ED interventions which was unable to determine whether interventions were superior to waitlist control groups [19]. It was found that computer-based interventions were effective, and more so for participants with binge/purge rather than restrictive ED symptoms. Thus, while the internet is a promising delivery modality for treatment of EDs, more research is required particularly involving a comparison with face-to-face delivery.

A systematic review evaluating the efficacy of internet-based interventions found that CBTgsh produced a significant improvement in primary and secondary outcomes related to eating behaviour and abstinence, across the six studies [76]. In addition, medium to high effect sizes were found within groups and between groups following utilisation of CBTgsh programs or a self-help book with supportive emails. The two studies utilising a specific writing task or email therapy not following a structured therapy program did not find any significant treatment effects.

A recent systematic review analysed the effectiveness of e-health interventions for the treatment of EDs [26]. The ED being investigated and the e-health intervention used varied across studies. Four studies reported higher effectiveness of e-health in comparison with control groups including waitlist, other psychotherapies, and TAU. A further systematic review found digital interventions to be more effective in reducing symptoms in prevention and established risk factors; however, few trials compared a digital intervention to a face-to-face intervention, limiting the conclusions that can be drawn [165].

High drop-out rates have been raised as a significant issue in internet-delivered treatments for EDs, with rates of dropout reported to be higher than that of face-to-face delivery, and an increased risk of drop-out being associated with more severe binge/purge and depressive symptoms [275]. These findings indicate that online interventions are better suited to individuals with less advanced EDs. For patients with BN who are motivated to change, iCBT may provide a more cost-effective intervention. Estimated cost per QALY was $59,540 (USD) compared with $73,618 for face-to-face CBT. This cost reduced further at 1-year follow-up to $38,715 per QALY for iCBT and $56,801 per QALY for face-to-face [285].

An RCT investigated the clinical efficacy of a combined mHealth intervention for EDs based on CBT and a mobile intervention through an application “TCApp” [28]. The study revealed that CBT can reduce ED symptoms regardless of its form of delivery. Another study examined the efficacy of a transdiagnostic cognitive-behavioural intervention for ED psychopathology delivered through a smartphone application [166]. Participants receiving the intervention reported greater reductions in EDE global psychopathology scores and ED symptoms, impairment and distress compared with the waitlist group.

A comparison of online CBT (CBT4BN) and face-to-face group CBT (CBTF2F) found online delivery to be inferior to face-to-face delivery at achieving binge/purge abstinence post-treatment [288]. However, this difference was no longer present at 12-month follow-up, indicating a ‘catch-up’ effect in the CBT4BN group. To address the issue of long waiting times experienced by patients with EDs between assessment and initial treatment session, Graham and Walton delivered a computer-based CBT intervention to outpatients with BN and BED prior to their first individual CBT program, finding it effective at reducing ED symptoms [101]; however, overall abstinence rates for both interventions were low at between 14 and 30% [101, 284]. Predictors for drop-out in this study included failure to engage, low perceived treatment credibility, previous experience with CBT, and randomisation to participant non-preferred format [284].

An RCT sought to investigate whether adding a brief online intervention, RecoveryMANTRA, which focuses on improving motivation to change and the development of a recover identity, would improve treatment outcomes for adults with AN [51]. No differences between the RecoveryMANTRA and TAU group were observed for BMI or ED symptoms. However, the RecoveryMANTRA group had significantly higher levels of confidence in own ability to change and therapeutic alliance with the therapist at the outpatient service, compared to TAU group without the online intervention [51].

In a systematic review, guided computer-based interventions (CBIs) for BN were found to lead to improvements in binge eating and purging behaviours, and global ED psychopathology, with those receiving guided CBIs improving more than controls and demonstrating similar efficacy to guided bibliography [83]. One study found CBIs to be potentially useful for relapse prevention of AN, but further empirical evidence is needed. Some studies suggest that CBIs may be an efficacious treatment option for adolescents with BN; however, more RCTs are required. Preliminary evidence suggested mobile interventions may be useful as an adjunct to therapy. Although most studies were able to demonstrate efficacy of CBIs, high rates of non-compliance, non-take-up and dropout across the included studies impede the validity of the results.

An RCT investigating the efficacy of a technology intervention delivered via text messaging after discharge from inpatient treatment for BN or OSFED found a significant difference between the intervention group (SMS-based maintenance intervention) and TAU in enhancing treatment outcomes [33]. An RCT conducted by Mitchell et al. [298] found CBT delivered face-to-face or via telemedicine to be equally efficacious in the treatment of in BN and OSFED. Taking this further, Ertelt et al. [80] sought to examine therapist and patient ratings of therapeutic alliance in both face-to-face and tele-delivery of CBT. Despite therapists indicating a stronger preference for face-to-face modalities and patients having no specific preference for treatment modality, ratings of perceived success and sustainability of treatment indicated strong therapeutic-relationships may be formed regardless of treatment delivery method [80].

##### Virtual reality

VR involves digital simulations of real-world situations where the participant is immersed in a scenario with visual and auditory stimuli [84]. It provides the opportunity to develop novel interventions to address body image and eating disturbance aspects of ED by simulating real world situations that would otherwise be difficult to replicate or control [55, 84–86, 173, 182, 203, 207].

A recent meta-analysis examining the efficacy of VR-enhanced CBT compared with CBT in the treatment of binge-purging type EDs found a significant reduction in binge frequency for those in the VR-enhanced CBT group compared with CBT group [173]. There were also significantly greater decreases in situation-induced body dissatisfaction in the VR-enhanced CBT group compared to CBT. However, there were no statistically significant differences in change in BMI or purge frequency, and no significant difference in improvement of overall body satisfaction [173]. Results highlight the possibility of VR in assisting patients in the development of coping strategies to triggers.

A randomised parallel study tested Cue Exposure Therapy (CET) in VR as an adjunct to CBT in individuals with BN and BED who required further treatment following their initial course [86]. CET in VR was superior to traditional CBT in terms of achieving binge/purge abstinence as well as reductions in core ED symptoms. Changes were maintained at follow-up, with abstinence rates remaining almost identical to post-treatment levels. These changes were well maintained in the same population group at 6-month follow-up, with abstinence rates remaining almost identical to post-treatment levels [85]. In a 1-year follow-up study of a VR-enhanced CBT intervention for treatment of body image disturbances in patients with ED, participants randomised to CBT + VR had a much faster response to CBT with improvements to ED psychopathologies maintained at follow-up [182].

A VR intervention targeting negative body memory in women with BED was found to be superior to CBT for weight loss in participants at 1-year follow-up [55]. A small (*n* = 38) RCT indicated that adjunctive therapy through use of a video game may improve treatment adherence in patients with BN [83]. VR was further investigated as a tool for reducing engagement in excessive exercising in patients with AN and BN, and was found to be effective at reducing urge to be physically active from levels measured at baseline [203].

### Carer/support interventions

Studies have shown that carers of patients with EDs can experience substantial distress, anxiety and depressive symptoms [63, 226, 241]; these are most prevalent among carers of individuals with AN [234, 241]. These symptoms have been shown to lead to maladaptive behaviours and contribute to disorder maintenance in the patient [63, 111], with higher perceived burden impacting successful implementation of therapy [186, 281].

Considering the complex and often enduring nature of EDs, there is an emerging evidence base regarding the importance of support for carers and a potential role for mentoring. Greater available support for individuals with EDs is associated with more positive outcomes for both carer and patient [91], and is particularly important considering the high amounts of perceived stigmatisation experienced by this group [63, 105, 183].

Interventions for carers such as online CBT, psycho-education and self-help programs, peer-mentoring and group workshops, have been found to be effective at reducing carer anxiety and depressive symptoms, and have been associated with improved treatment outcomes for patients [111, 121, 125, 128, 205, 206, 216]. The Collaborative Care Skills workshop [234], developed in the UK, and delivered to Australian carers in two studies, reported a significant reduction in maladaptive coping and expressed emotions [206]. This was maintained at 8-week and 6-month follow-up, along with an increased confidence that their loved ones could change, which was considered a contributor to measured decreases in psychological distress.

## Discussion

The primary aim of the RR was to identify, summarise and consolidate the evidence-base for psychotherapies and their effectiveness in the treatment of EDs. The secondary aim was to identify gaps in the research and highlight emerging psychotherapeutic treatments. In total, 263 studies met broad eligibility criteria. Of these, 30% focused on CBT, 12% on FBT, 5% on exposure-based therapies, 3% on IPT, DBT, ACT, and ICAT, and 2% on other psychotherapies. CBT was considerably the most widely researched, and behavioural therapies in general comprised at least two-thirds of the examined literature.

Much of the research examining CBT and its variations has focused on the treatment of BN, BED, and AN, and it is currently suggested to be the leading treatment for individuals with BN and BED. There is general consensus in the field regarding the transdiagnostic therapeutic effectiveness of CBT across different ED diagnoses given its ability to target illness-maintaining features and reduce binge/purge symptomatology [69, 167], although the evidence is greater for use in BN and BED as well as their sub-clinical variants. Moreover, emerging evidence suggests that CBT may be effective when administered using group therapy [24, 66, 134], guided self-help [46, 89, 273, 279] and technology-based delivery modalities [52, 76, 229, 250, 255, 280, 297]. Despite the high number of included studies, few investigated the efficacy of CBT for OSFED and ARFID.

Currently, FBT should be considered in the first instance when treating children and adolescents with AN (including atypical presentations, OSFED/UFED) [100]. It is the most consistently effective treatment for child and adolescent AN and has been found to be highly cost-effective in an Australian context [20, 157]. However, non-response to treatment and high drop-out rates have resulted in several adaptive variants to the intervention, including multi-family, parent-focused and systematic family therapies, with varying degrees of effectiveness. Whilst much less evidence exists on the use of FBT to treat adolescent BN than AN, there is a developing evidence-base for treatment of BN and BED with FBT [159]. Factors associated with higher remission rates include sustained involvement by fathers in therapy and early weight gain in response to treatment [130]. Preliminary evidence suggests that FBT may be a viable option to treat ARFID in children and adolescents [259].

IPT has been considered an effective and viable alternative treatment for BN and BED, however, opinions in the field regarding its inferiority to CBT in being able to produce a rapid treatment response may have contributed to a lack of research on the intervention [81]. IPT has demonstrated similar treatment outcomes to both individual and group CBT. Most improvements from IPT have been observed at follow-up, suggesting its efficacy in long-term reductions and remission of symptoms [195, 230, 295, 299]. IPT for the prevention of excess weight gain may be efficacious for reducing loss of control eating and weight gain in overweight youth [223].

There is preliminary evidence to suggest therapies involving exposure to stimuli eliciting ED behaviours and the subsequent restructuring of these responses could be helpful in increasing long-term effectiveness when delivered as a supplement to CBT [104, 245, 253]. Pharmacotherapy may enhance the effects of exposure therapy in individuals with AN [161].

Novel psychotherapeutic approaches as an alternative to CBT are being trialled in RCTs [208, 287]. From the small number of studies identified, neither ICAT or ACT have proven to be more effective than an active comparator (CBT) or TAU for patients with AN or BN. However, emerging evidence suggests that ICAT may be equally as effective as CBT-E and guided self-help at reducing symptoms of bulimia nervosa [208, 287].

Overall, the available evidence for psychotherapies delivered in a group format suggests that the type of therapy rather than its delivery in individual or group settings contributes more to the effectiveness of the intervention. Although there is some evidence to suggest that MFT may be more effective than standard FBT in producing changes on outcomes due to its networking and support aspects, evidence for the advantages of group over individual for other types of psychotherapies is inconclusive. On the other hand, the available evidence does not appear to show dilution of therapeutic effect when delivering psychotherapy interventions to groups, and this alternative modality may have significantly reduced resource implications.

Research suggests GSH as the first-line treatment for non-underweight EDs characterised by recurrent binge eating, namely BED and BN [208]. Evidence for its effectiveness on individuals with BED is particularly strong [53, 243, 265, 286]. Self-help interventions are not recommended in the treatment of AN due to the specialist care required for this disorder [286]. It has been suggested that the comparative advantages of self-help over therapist-led interventions include increased access, cost-effectiveness and capacity to be delivered by professionals without specialist knowledge in ED treatment [175, 286].

Studies have found technology-based CBT interventions to result in significant reductions in ED symptoms compared with controls among individuals with BN, BED and OSFED [173, 255]. At 4-month follow-up iCBT with GSH was found to be inferior to individual face-to-face CBT in reducing binge eating in patients with BED [33, 74]. However, no differences between online and face-to-face interventions were reported at 18-month follow-up, suggesting that iCBT with GSH—which may be easier to access and more affordable—may be a viable alternative over longer-term face-to-face therapy [33, 74]. High drop-out rates have been raised as a significant issue in internet-delivered treatments for EDs [155, 275]. These findings indicate that online interventions may be better suited to individuals with less advanced trajectories of EDs.

This review has highlighted that CBT delivered face-to-face or via telemedicine to BN or OSFED patients has been found to be equally efficacious in terms of treatment outcomes. Therapists tend to favour face-to-face CBT over tele-delivery, however patients do not show a strong preference for either delivery modality with little to no impact on the therapeutic alliance [80].

Overall, the evidence supports the augmentation of CBT with VR to increase effectiveness and time to response in individuals with ED [173]. However, the practicality of providing these novel interventions must be considered, as it is unlikely that many therapists would currently have or could gain the relevant expertise to deliver VR-based therapy.

Evidence is limited in terms of ED carer support, nonetheless, there is a growing need to support individuals caring for people with an ED [93, 111]. Further research and investment in mentoring programs for carers of someone with an ED may be warranted as part of a ‘stepped care’ approach, delivered in a similar way to mental health services in Australia, however, evidence on effect is still in its infancy.

There is very limited evidence addressing treatment for ARFID. Available literature consists of case reports and case series. Some evidence exists for ‘feeding disorder of infancy and early childhood,’ a DSM-IV disorder now encompassed by ARFID. However, these lack applicability to adolescents and adults.

### Strengths and limitations

Inherent limitations associated with the RR methodology aside, the current review faced a number of unique challenges. Firstly, the broad eligibility criteria, particularly for the large topic of psychotherapies, meant that a significant number of identified studies focused on small variations of the same core psychotherapy; thus, lacking diversity in ED treatment types. For example, several studies investigated versions of CBT, such as CBT-L, and CBT-S, where the core tenets of the psychotherapeutic approach were much the same as the therapy they were based on. Secondly, there appeared to be a large proportion of literature that was not captured or was considered ‘ineligible’ for inclusion, which may have led to a skewed representation of the evidence-base related to specific therapies. Finally, much of the literature examined for the RR centred on clinical samples in Westernised, Educated, Industrialised, Rich, and Democratic (WEIRD) countries. This may suggest a bias in the research and may limit the generalisability of the findings and conclusions of the RR to countries and populations not captured in the studies.

Despite these limitations, this review enabled a broad investigation into the current evidence-base whilst highlighting areas that are lacking research. Further, it offers a springboard for clinical and academic discussion to move the field forward and improve clinical outcomes and care.

### Future studies

Considerably more research is needed to critically and meaningfully consider other third-wave therapies that may be able to demonstrate greater therapeutic outcomes over traditional psychotherapies, such as CBT [162]. CBT has emerged as the most empirically researched psychotherapy, yet it has not been able to demonstrate greater propensity to remit EDs. Specifically, non-response to treatment in some groups, such as AN, and lack of improvement in remission rates have necessitated exploration of alternative approaches. Future research into alternatives which consider complex presentations and treatments that address both the high dropout rates, such as those seen in FBT for children and adolescents, would greatly benefit the field of EDs. Additionally, variations in outcome measurements may impact comparability of studies and the ability for appropriate evaluation of treatment effectiveness across psychotherapies. Lastly, investigation into treatments that focus on emerging disorders, notably ARFID and OSFED, would aid in supporting patients, families, and clinicians by addressing the paucity in evidence-based interventions available for these types of EDs.

## Conclusions

The current evidence suggests that a number of psychotherapies are effective in improving ED symptomatology. Specifically, interventions based on cognitive-behavioural approaches, such as CBT, FBT, and IPT, have the most demonstratable evidence supporting their efficacy as treatments for EDs. The RR indicated that there is emerging evidence for novel approaches including ACT, DBT, cognitive remediation therapy (CRT), and exposure and response (ERP) therapies demonstrating varying efficacy. It also highlighted emerging evidence supporting technology-based delivery of psychotherapies. Finally, this review has served to highlight the notion that every treatment approach is subject to its strengths and shortcomings and given the complexity of EDs and the influence of individual factors, there is very unlikely to ever be a ‘one-size fits all’ treatment. Nonetheless, this review starkly highlights that more needs to be done in order to improve ED outcomes, and related impacts and burden.

## Data Availability

Not applicable—all citations provided.

## Abbreviations

AAT: Appetite Awareness Therapy
ABM: Approach bias modification
ACT: Acceptance and commitment therapy
AFT: Adolescent-focused individual therapy
AN: Anorexia nervosa
AN-BP: Anorexia nervosa binge/purge subtype
AN-BP: Anorexia nervosa-borderline personality
AN-EXRP: Exposure response therapy for anorexia nervosa
AN-R: Anorexia nervosa restrictive subtype
ARFID: Avoidant restrictive food intake disorder
BED: Binge eating disorder
BFST: Behavioural family system therapy
BMI: Body mass index
BN: Bulimia nervosa
BPD: Borderline personality disorder
BT-BN: Bulimia nervosa-focused family therapy
BWLT: Behavioural weight loss therapy
CALD: Culturally and Linguistically Diverse
CBI: Computer based intervention
CBT: Cognitive behavioural therapy
CBT-A: Appetite-focused cognitive behavioural therapy
CBT-A: Cognitive behavioural therapy for adolescents
CBT-BN: Cognitive behavioural therapy for bulimia nervosa
CBT-E: Enhanced cognitive behavioural therapy
CBTgsh: Cognitive behavioural therapy guided self-help
CBT-L: Cognitive behavioural therapy-long
CBT-S: Cognitive behavioural therapy-short
CET: Cue exposure therapy
CFT: Conjoint family therapy
CRT: Cognitive remediation therapy
CRT: Cognitive remediation therapy
DBT: Dialectical behavioural therapy
DBT-BED: Dialectical behavioural therapy for binge eating disorder
DBT-AF: Appetite focused dialectical behavioural therapy
ED: Eating disorder
EDE: Eating disorder examination
EDE-Q: Eating disorder examination questionnaire
EDNOS: Eating disorder not otherwise specified
EFTG: Emotionally-focused group therapy
eGSH: Email guided self-help
ERP: Exposure response therapy
FBT: Family based therapy
FBT-AN: Family based therapy for anorexia nervosa
FBT-BN: Family based therapy for bulimia nervosa
FPT: Focal psychodynamic therapy
FT-AN: Family based therapy for anorexia nervosa
GSH: Guided self-help
HAPIFED: Health approach to weight management and food in eating disorders
HRQoL: Health-related quality of life
ICAT: Integrative cognitive affective therapy
ICAT-BED: Integrative cognitive affective therapy for binge eating disorder
ICAT-BN: Integrative cognitive affective therapy for bulimia nervosa
iCBT: Online delivery of cognitive behavioural therapy
IPT: Interpersonal therapy
LOC: Loss of control
MANTRA: Maudsley anorexia treatment for adults
MET: Motivational enhancement therapy
MFT-AN: Multi-family therapy for anorexia nervosa
MI: Motivational interviewing
MOPED: Motivation and psycho-educational guided self-help intervention for people with eating disorders
NED: Night Eating Syndrome
NHS: National Health Service
NICE: National Institute for Clinical Excellence
OSFED: Other Specified Feeding and Eating Disorder
PDT: Psychodynamic therapy
PFT: Parent focused therapy
PPT: Positive psychology
RCT: Randomised clinical trial
RR: Rapid review
S-BED: Subclinical binge eating disorder
S-BN: Subclinical Bulimia Nervosa
SPACE-ARFID: Supportive parenting for anxious childhood emotions adapted for avoidant/restrictive food intake disorder
SyFT: Systemic family therapy
TAU: Treatment as usual
UFED: Unspecified feeding or eating disorder
VR: Virtual reality
WEIRD: Westernised, educated, industrialised, rich, democratic (countries)

## Footnotes

1 *A sham procedure without active neuro modulation.*
